# An Overview of 1,2,3-triazole-Containing Hybrids and Their Potential Anticholinesterase Activities

**DOI:** 10.3390/ph16020179

**Published:** 2023-01-24

**Authors:** Shah Alam Khan, Mohammad Jawaid Akhtar, Urvashee Gogoi, Dhanalekshmi Unnikrishnan Meenakshi, Aparoop Das

**Affiliations:** 1 College of Pharmacy, National University of Science and Technology, Muscat 130, Oman; 2 Department of Pharmaceutical Sciences, Dibrugarh University, Dibrugarh 786004, India

**Keywords:** acetylcholine, acetylcholinesterase, Alzheimer’s disease, butyrylcholinesterase, hybrids, 1,2,3-triazole

## Abstract

Acetylcholine (ACh) neurotransmitter of the cholinergic system in the brain is involved in learning, memory, stress responses, and cognitive functioning. It is hydrolyzed into choline and acetic acid by two key cholinesterase enzymes, viz., acetylcholinesterase (AChE) and butyrylcholinesterase (BuChE). A loss or degeneration of cholinergic neurons that leads to a reduction in ACh levels is considered a significant contributing factor in the development of neurodegenerative diseases (NDs) such as Alzheimer’s disease (AD). Numerous studies have shown that cholinesterase inhibitors can raise the level of ACh and, therefore, enhance people’s quality of life, and, at the very least, it can temporarily lessen the symptoms of NDs. 1,2,3-triazole, a five-membered heterocyclic ring, is a privileged moiety, that is, a central scaffold, and is capable of interacting with a variety of receptors and enzymes to exhibit a broad range of important biological activities. Recently, it has been clubbed with other pharmacophoric fragments/molecules in hope of obtaining potent and selective AChE and/or BuChE inhibitors. The present updated review succinctly summarizes the different synthetic strategies used to synthesize the 1,2,3-triazole moiety. It also highlights the anticholinesterase potential of various 1,2,3-triazole di/trihybrids reported in the past seven years (2015–2022), including a rationale for hybridization and with an emphasis on their structural features for the development and optimization of cholinesterase inhibitors to treat NDs.

## 1. Introduction

Acetylcholine (ACh), a neurotransmitter produced by cholinergic neurons, is linked to signal transduction pathways involved in memory, motivation, behavioral adaptability, associative learning, sensory perception, and motor control [1]. A key step in the recovery of the cholinergic neuron is the hydrolysis of the neurotransmitter ACh into choline and acetic acid by a family of enzymes known as cholinesterases (ChE). The two kinds of ChE are acetylcholinesterase (AChE; EC 3.1.1.7) and butyrylcholinesterase (BuChE; EC 3.1.1.8). Both AChE and BuChE are serine esterases because they include a serine amino acid residue, which is required for catalytic activity [2].

AChE, also referred to as true/erythrocyte ChE, is one of the most efficient cholinergic neurotransmission enzymes. AChE is often produced or expressed by muscles, neurons, and some hematopoietic cells [1]. AChE found in the neuromuscular junction (NMJ) of skeletal muscle is produced by the muscle, not the nerve cell. Compared with AChE, BuChE is not as well known for its relevance. The ability of AChE to preferentially hydrolyze Ach over butyrylcholine distinguishes it from BuChE. AChE exists in a variety of molecular forms, all of which have comparable catalytic properties but differ in terms of how they oligomerize and how they adhere to the surface of cells [3]. BuChE, also known as pseudo/plasmatic ChE, is exported into the plasma after being generated in the liver. It is thought to be crucial for the metabolism of choline and non-choline esters such as cocaine, heroin, succinylcholine, butyrylcholine, and ACh. Compared with AChE, it has a wider substrate range for catalysis. BuChE is recognized as regulating cholinergic neurotransmission and the expression of a specific population of neurons [4].

**Figure 1:**
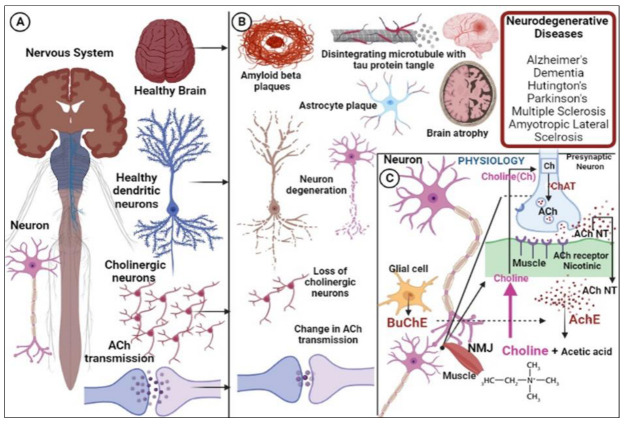
(**A**) Schematic illustration of a healthy brain and nervous system with a focus on cholinergic neurons and circuits in the general population. (**B**) Alterations/changes in cholinergic function and related pathologies spurred by those changes in neurons and neuronal circuits. (**C**) ACh’s function at synapses: The primary precursor for the synthesis of Ach is choline. Choline and Acetyl-CoA are combined to create Ach by the action of choline acetyltransferase (ChAT). ACh controls cholinergic signaling in various parts of the brain and binds to nicotinic or muscarinic receptors on postsynaptic neurons/tissues, including muscles (NMJ). The NMJ’s primary function is to translate the temporal sequence of motor neuron action potentials (APs) into muscular contractions. The activity of AChE causes extra ACh at synapses to be converted to choline and acetic acid. BuChE found in the glial cells is also involved in ACh metabolism. Transporters are then used to recycle choline back to the neuron and nerve terminal for further release.

Cholinergic neurons play a crucial role in maintaining the balance between excitation and inhibition in neural circuits and fine-tuning brain activity. ACh neurotransmitter (NT), must be produced and released for cholinergic transmission to occur. The enzyme choline acetyltransferase (ChAT) transfers the acetyl group from acetyl-coenzyme A to choline and is the single step in the production of ACh. ACh has different effects on neuronal signaling depending on its location of release, an affinity for particular cholinergic receptor types (nicotinic or muscarinic receptors), its removal from the synaptic end by AchE, and the type of neuronal targets. Figure 1 illustrates the pathologies of the alteration in the cholinergic functions and actions of ChEs.

ACh is known as a neuromodulator in the nervous system because it can produce activation or inhibition in a neuron’s firing based on the sort of external stimuli or inputs that the target neuron receives [5]. Because the majority of the brain regions (cerebral neocortex, hippocampus, basal forebrain, and the nucleus basalis of Meynert) innervated by cholinergic neurons, these are involved in learning, memory, stress responses, and cognitive functioning. The degeneration of these cholinergic neurons is regarded as a significant contributing factor in neurodegenerative disorders. It has been demonstrated that the key indicators of cholinergic neuronal activity, ChAT and AChE levels, are impacted in neurodegenerative disorders (NDs) [6]. It has become increasingly evident that the health of numerous interdependent neural circuits, notably striatal cholinergic interneurons (CINs), tightly controls mammalian brain processes, from executive and motor functioning to memory and emotional reactions. The cholinergic innervation of subcortical regions, which originates in the basal forebrain and brainstem, is crucial to orchestrating both cognitive and non-cognitive symptoms in Parkinson’s disease (PD), Alzheimer’s disease (AD), and other NDs [7,8]. There is also an established link between the cholinergic system and neuropsychiatric disorders [1]. ChAT activity was found to be reduced in the hippocampus, cortex, and amygdala of AD patients [8]. The peculiar pharmacology of AChE is seen in neurogenesis, cell adhesion, synaptogenesis, and the activation of dopamine neurons, as well as in the assembly of amyloid beta (Aβ) fibers and the control of glutamate-mediated hippocampal activity. Its sudden blocking has catastrophic repercussions for AChE. Nerve agents and insecticides, which can irreversibly inhibit enzymes, belong to a family of very toxic substances frequently exhibit this effect. BuChE tangled in neurogenesis has a reclaiming impact on several xenobiotic substances and is crucial for cholinergic mediation. Pharmacologically, it is also believed that when AChE malfunctions, BuChE takes control [9]. According to studies, BuChE activity is greatly increased (41–80%) in the brains of people with advanced AD, particularly in areas affected by Aβ plaques and neurofibrillary tangles (NFT), in contrast to a 10–60% AChE deficit [10]. The fact that BuChE has a longer half-life than AChE and has more glycation sites, which are crucial for ChE stability and clearance, suggests that BuChE may be a possible target in the treatment of NDs, particularly AD. As an alternative to AChE, BuChE regulates ACh hydrolysis, and therapeutic drugs that inhibit both AChE and BuChE may be more advantageous for treating NDs than those that only inhibit AChE [11]. There is widespread consensus that reversible inhibitors that target ChE can enhance people’s quality of life and, at the very least, temporarily lessen the symptoms of NDs [12]. Based on the abovementioned details, both ChEs are regarded as extremely pertinent targets in the drug discovery and development process for NDs. Novel ChE inhibitors have been the focus of numerous research teams. Recently, it was discovered that many families of heterocyclic compounds, such as 1,2,3-triazoles, indole, coumarin, quinolones, etc., potently inhibit both AChE and BuChE [13,14].

The 1,2,3-triazole heterocycle present in drugs exhibits antifungal, antibiotic, anticancer, antiviral, antimigraine, and anticonvulsant activities, as shown in Table 1. The 1,2,3-triazole ring produces anti-ChE activity by inhibiting both AChE and BuChE activities. The 1,2,3-triazole ring possesses low multidrug resistance, low toxicity, high bioavailability, and stability in both acidic and basic conditions. The nitrogen atom in 1,2,3-triazole ring is responsible for the enzyme–inhibitor interaction [15]. Despite the importance of 1,2,3-triazole, with its wide range of biological activities, there is still a need for the development of novel, multitargeted inhibitors of ChE enzymes for ND treatment. This review covers an overview of the synthesis of 1,2,3-triazole derivatives with some in silico interaction, important for the anti-ChE activity reported recently by various researchers.

## 2. Chemistry and the Importance of the Triazole Ring System

**Figure 2:**
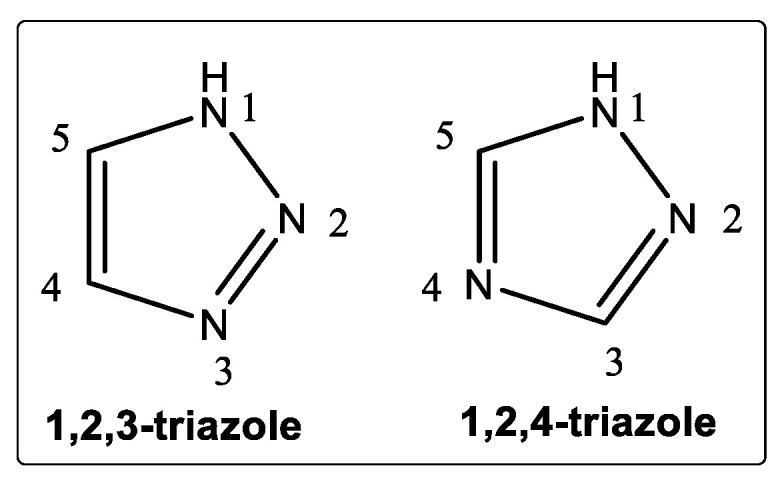
Isomers of triazole.

Triazoles are an important group of nitrogen-containing heterocyclic scaffolds containing two carbon atoms and three nitrogen atoms in an aromatic, five-membered ring framework [16]. Alternatively known as pyrrodiazoles, they are di-unsaturated rings composed of three nitrogen atoms within a heterocyclic core, with isomeric forms of 1,2,3-triazoles and 1,2,4-triazoles [17,18]. In chemical terms, a triazole is one of two isomeric compounds (Figure 2**)** with the molecular formula C_2_H_3_N_3_.

Several drugs and pharmaceutical agents contain triazoles as their core structure. In addition to being structurally and chemically diverse, triazole-based molecules, as central scaffolds, are capable of interacting with a variety of receptors and enzymes, showing broad biological activities [19,20]. As a result, a wide range of triazole-containing compounds has been frequently employed as clinical drugs or candidates, and they are vital for treating a wide variety of diseases, including antibacterial [21], antifungal [22], antitubercular [23], antiviral [24], anti-Alzheimer’s [25], anticancer [26], antiparasitic [27], and antidiabetic treatments [28]. On the global market, triazole ring-containing drugs with medicinal value are actively exploited. Table 1 lists several pharmacological actions and chemical structures of triazole-based compounds.

### 2.1. Various Synthetic Routes for the Synthesis of 1,2,3-triazole Scaffold

Numerous innovative techniques for 1,2,3-triazole synthesis have been created up to this point, including Watson cycloaddition [29], later updated as Huisgen 1,3-dipolar cycloaddition [30]; metal-catalyzed 1,3-dipolar cycloaddition [31]; strain-promoted azide–alkyne cycloaddition [32], the metal-free synthesis of 1,2,3-triazoles [29]; and so on.

#### 2.1.1. Huisgen 1,3-dipolar Cycloaddition

**Figure 3:**
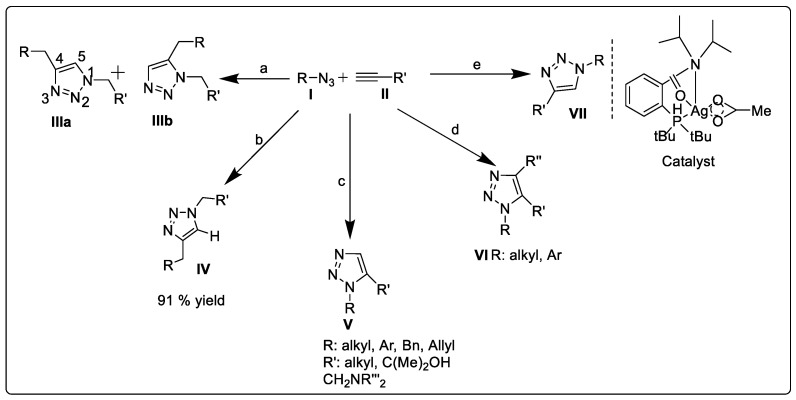
Synthesis of 1,2,3-triazoles: (a) 18 h, 98 °C; (b) CuSO_4_.5H_2_O (1 mol%), sodium ascorbate (5 mol %), H_2_O/t-BuOH (2:1),25 °C, 8 h; (c) 2 mol-%, Cp*RuCl(PPh_3_)_2_, dioxan, 60 °C, 12 h; (d) 2 mol-%, Cp*RuCl(COD), toluene, RT, 30 min; (e) catalyst (2–2.5 mol %), caprylic acid (20 mol%), toluene, 24 h, 90 °C.

From the 1950s to 1970s, Huisgen explored the 1,3-dipolar cycloaddition of alkyne and azide (Figure 3) as a method of synthesizing 1,2,3-triazoles [29]. A Huisgen cycloaddition results from the reaction of a dipolarophile with a 1,3-dipolar compound to produce five-membered (hetero)cycles [33].

Azide **II**, upon reaction with alkyne **I**, yields 1,2,3-triazole as a mixture of 1,4-adduct **IIIa** and 1,5-adduct **IIIb** after 18 h at 98 °C. In typical synthetic conditions, azides are preferred due to their relative stability and lack of side reactions. Another research group modified Huisgen cycloaddition into a more regioselective, copper-catalyzed stepwise reaction [34].

#### 2.1.2. Copper-Catalyzed Azide–Alkyne Cycloaddition (Click Chemistry)

**Figure 4:**
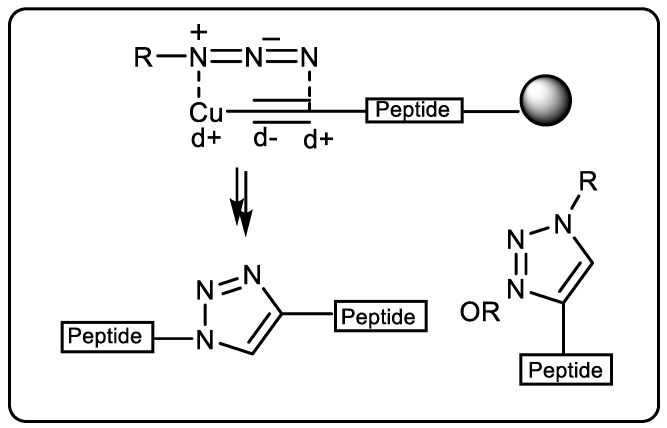
Copper(I)-catalyzed 1,3-dipolar cycloaddition of alkynes to azides affording peptidotriazoles or *N*-substituted histidine analogs.

“Click chemistry” was coined by KB Sharpless in 2001 to describe reactions that are high-yielding and wide-ranging, contain only by-products that can be eliminated by chromatography, are stereospecific, are easy to perform, and can be conducted in solvents that are easily removed or benign [35]. Huisgen cycloadditions of organoazides and alkynes at high temperatures proceed slowly, resulting in 1,4- and 1,5-disubstituted 1,2,3-triazoles. Copper-catalyzed azide–alkyne cycloadditions (CuAAC) were independently discovered by Meldal and Sharpless in 2002 [36]. The terminal alkyne substrate is attached to a hydrophilic tertiary amide-poly(ethylene glycol)-based resin using a peptide linker in Meldal’s method (Figure 4). The corresponding 1,2,3-triazole is produced when the azide is added in mild conditions using copper(I) salts as catalysts [37,38]. The 1,4-disubstituted 1,2,3-triazole is the only product of this reaction, which proceeds at room temperature in a variety of organic solvents with quantitative conversion [39].

On the other hand, the Sharpless group identified a solution-phase, copper-catalyzed azide–alkyne cycloaddition (Figure 3). In their typical process, ascorbic acid or sodium ascorbate reduces the economically advantageous salt copper(II) sulfate pentahydrate in situ in a solvent mixture of water and alcohol (“Sharpless–Fokin conditions”). In contrast to Huisgen’s uncatalyzed method, this strictly regioselective stepwise process only produces 1,4-disubstituted 1,2,3-triazole (**IV**) and speeds up the reaction by up to 107 times [40] (Figure 3).

#### 2.1.3. Ruthenium-Catalyzed Azide–Alkyne Cycloaddition (RuAAC)

The Boren group made more excellent discoveries in 2005 with regard to the regioselective synthesis of 1,5-disubstituted-1,2,3-triazoles [41]. This new approach (Figure 3) uses Ru(II)-catalysts to regioselectively direct the 1,3-dipolar cycloaddition of azides to terminal alkynes (RuAAC), resulting in 1,5-disubstituted 1,2,3-triazole (**V**) as the principal product. Additionally, Cu-catalysis only allowed for the cycloaddition of terminal alkynes, whereas Ru(II) complexes supported both internal and external cycloadditions (**VI**) of alkynes with organic azides [41].

#### 2.1.4. Silver-Catalyzed Azide–Alkyne Cycloaddition (AgAAC)

In 2011, McNulty and coworkers pioneered the use of silver in the field of click chemistry (primarily in AAC reactions) [42]. They described the creation of a novel silver(I) acetate complex and located a well-defined species that catalyzed the first common pure silver azide–alkyne cycloaddition (Ag-AAC) reaction (Figure 3). Silver acetate was treated in a 1:1 ratio with the ligand 2-(di-*tert*-butylphosphino)-*N*,*N*-diisopropylbenzamide to create the chosen catalyst. Using only 2–2.5 mol% of this catalyst at 90 °C, phenylacetylene and a slight excess of benzyl azide react to produce 1,4-triazole isomer (**VII**) in a greater than 99% yield [43].

#### 2.1.5. Strain-Promoted Azide–Alkyne Cycloaddition

**Figure 5:**
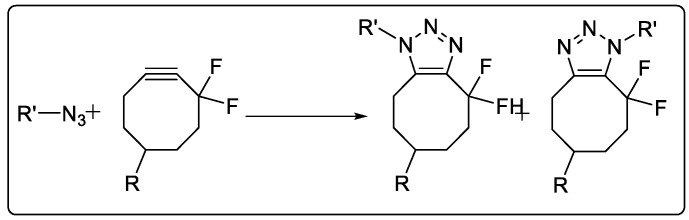
Strain-promoted [3 + 2] cycloaddition of azides and cyclooctynes.

Agard et al. investigated a fascinating strain-promoted azide–alkyne cycloaddition (SPAAC) chemical procedure for bioconjugation [44]. Rather than employing Cu(I) to activate the alkyne, the alkyne is placed into a strained difluorooctyne (DIFO), where the electron-withdrawing, propargylic, gem-fluorines work in conjunction with the ring strain to significantly destabilize the alkyne [45] (Figure 5).

#### 2.1.6. Metal-Free Synthesis of 1,2,3-triazoles

With the incredible advancements in 1,2,3-triazole synthesis, the development of a metal-and azide-free technique has emerged as a fairly significant problem from the perspectives of economics and green chemistry.

##### Synthesis of 1,2,3-triazole through α,α-dichlorotosylhydrazones

**Figure 6:**
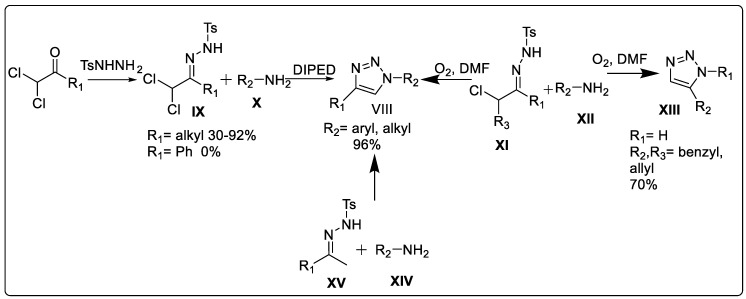
Metal-free methods of synthesizing 1,2,3-triazole.

A revolutionary work toward the metal-free, azide-free synthesis of 1,2,3-triazole **VIII** was reported by Sakai et al. [46] as early as 1986 by condensing α-dichlorotosylhydrazone **IX** with primary amine **X** under ambient conditions (Figure 6). In 2012, van Berkel et al. [47] perfectly exploited the mechanism, scope, and limitations of the Sakai reaction to demonstrate that this transformation is suitable as a scheme for metal- and azide-free 1,2,3-triazole formation.

##### Synthesis of 1,2,3-triazole through α-chlorotosylhydrazones

Bai et al.’s [48] modification of the Sakai reaction substituted α-chlorotosylhydrazones **XI** in place of α,α-dichlorotosylhydrazones to produce 1,2,3-triazole. Via the cycloaddition of α-chlorotosylhydrazones with arylamines **XII** in a metal-free and azide-free environment, this synthetic approach provides a broad path toward the synthesis of 1,4-disubstituted **VIII** and 1,5-disubstituted 1,2,3-triazole **XIII** (Figure 6).

##### Synthesis of 1,2,3-triazole through N-tosylhydrazones

In their proposal, Cai et al. [49] suggested using aniline **XIV** to catalyze the oxidative formal [4+1] cycloaddition of *N*-tosylhydrazone **XV** with 1,4-disubstituted-1,2,3-triazole **VIII** (Figure 6). The suggested synthetic method uses the I_2_/TBPB system as an alternative catalyst in place of heavy metals. To produce 1,4-disubstituted-1,2,3-triazoles, this single-operation technique involves functionalizing the C(sp^3^)-H link, forming the N-N/C-N bonds, and cleaving the S-N bond.

#### 2.1.7. Ionic Liquid-Catalyzed Synthesis

**Figure 7:**
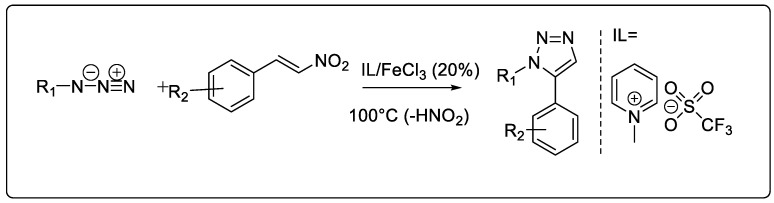
Choline chloride-CuCl-catalyzed Huisgen cycloaddition.

Ionic liquids (IL) have special qualities that make them effective solvents for a variety of organic, inorganic, and polymeric compounds. These properties include low vapor pressure, a large liquid range, great chemical stability, high thermal stability, and strong solvent power [50]. Choline chloride-CuCl, a straightforward bifunctional IL catalyst, was discovered to be extremely active for [3 + 2] Huisgen cycloaddition in H_2_O [51] (Figure 7).

### 2.2. Application of Triazoles in the Synthesis of Other Heterocyclic Compounds

The presence of three nitrogen atoms in triazole structures has provided possibilities for a wide variety of structural alterations, leading to development of novel, therapeutically promising medicines.

Currently, the most commonly prescribed antifungals in clinical therapy are triazole drugs (fluconazole, itraconazole, voriconazole, and posaconazole). Recent studies on triazoles have utilized the principles of conjugate chemistry to assemble active pharmacophores to produce hybrid molecules with the desired activities. Triazoles clubbed with other pharmacophores have been found to have a broader spectrum with antimicrobial [52], anti-inflammatory [53], antineoplastic [26], antiobesity [54], antidiabetic [28], immunomodulatory [55], anticholinesterase [56], and antiviral activities [24]. In addition to the available triazole drugs, researchers are interested in exploring and developing new triazole-based scaffolds with major applications in biomedical and biotechnology fields.

## 3. Conjugates of 1,2,3-triazole as Potential AChE and BuChE Inhibitors

### 3.1. Hesperetin–1,2,3-triazole Hybrids

**Figure 8:**
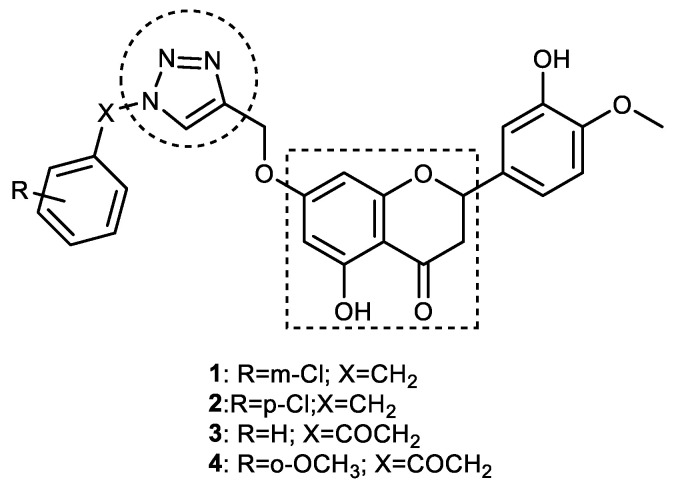
Chemical structure of hesperetin–1,2,3-triazole hybrids.

Hesperetin, a 4′-methoxyflavanone, is one of the most important bioactive phytochemicals present in citrus fruits. It has been reported to exhibit a wide spectrum of pharmacological activities, including anti-ChE inhibitory activity [57]. To improve its anti-ChE, anti-neuroinflammatory, and neuroprotective repertoire, hesperetin was clubbed with a 1,2,3-triazole motif to obtain 39 new compounds belonging to 4 series of 7-O-1,2,3-triazole–hesperetin hybrids. Interestingly, all the synthesized hybrid compounds showed better AChE inhibitory activity in comparison with the parent flavanone, hesperetin (IC_50_ = 3.04 ± 0.21 μM). Furthermore, 7-O-amide hesperetin derivatives displayed almost twofold greater BuChE inhibitory activity than 7-O-triazole derivatives. Among all the hybrids, four compounds, **1**, **2**, **3**, and **4** (IC_50_ = 3.08, 4.61, 6.37, and 5.51 μM, respectively), were found to be more potent than donepezil (IC_50_ = 6.21 ± 0.52 μM) in BuChE inhibition (Figure 8). Compound **1** (7-O-((1-(3-chlorobenzyl)-1*H*-1,2,3-triazol-4-yl) methyl) hesperetin), the most potent anti-BuChE compound (IC_50_ = 3.08 ± 0.29 μM), did not show any sign of neurotoxicity (Aβ-induced SH-SY5Y) in concentrations up to 50 μM and showed good blood–brain barrier (BBB) permeability along with notable neuroprotective and anti-neuro-inflammatory activities (IC_50_ = 2.91 ± 0.47 μM) against NO production. Mechanistic studies revealed that the anti-inflammatory activity of compound **1** could be due to its ability to block the NF-κB signaling pathway via the inhibition of the phosphorylation of the P65 protein. Additionally, it was able to inhibit Aβ_1–42_ aggregation, decreased the generation of ROS, and could selectively chelate biometals such as Cu^2+^, which all contribute to its neuroprotective activity. The administration of compound **1** to scopolamine-induced AD mice led to significant improvement in learning and memory impairment. Enzyme kinetic studies indicated compound **1** to be a mixed-type inhibitor of BuChE. The strong anti-BuChE activity of compound **1** was probed via molecular docking studies on BuChE (PDB code: 5NN0), which revealed strong binding to the active site on the target protein. The ketone of the pyrone ring interacted with Tyr128 to form one hydrogen bond, while ring A (benzene) of the flavanone formed hydrophobic interactions (π-sigma) with Trp82, Ala328, and His438 in the active pocket of the receptor [58]. A 1,2,3-triazole-ring-bearing chlorobenzyl substituent formed a π–anion interaction with Asp70. Based on these results, compound **1** could be considered a potential MTDL candidate in the development of new anti-AD agents [59].

### 3.2. Genipin–1,2,3-triazole Hybrids

**Figure 9:**
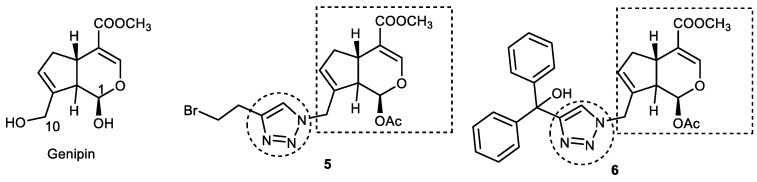
Chemical structures of genipin and genipin–1,2,3-triazole hybrids.

Geniposide (an iridoid glycoside) is a major component of the fruit of *Gardenia jasminoides* Ellis, which, upon hydrolysis, yields biologically active genipin aglycone. Genipin shows potential neuroprotective activity by virtue of its ability to inhibit high-level lactate dehydrogenase (LDH) in the blood, thereby preventing amyloid-β (Aβ) peptide toxicity [60]. Encouraged by the anti-AChE and Aβ_1–42_ aggregation inhibitory activity of piperazine–genipin hybrids [61], Silalai et al. prepared genipin–1,2,3-triazole hybrids in search of anti-AD agents. A total of 39 compounds were synthesized, which were evaluated for AChE and BuChE inhibitory activity in addition to neuroprotective action against hydrogen peroxide-induced neuronal toxicity. Intermediate genipin azides were reacted with different alkynes to undergo azide−alkyne Huisgen cycloaddition reactions using copper iodide to obtain C-10-substituted genipin–1,2,3-triazole derivatives in good-to-excellent yields. Substituted 1,2,3-triazoles with different carbon chain lengths, such as phenyl, benzyl ether, and benzylamine, as well as aliphatic, phthalimide, and alicyclic functionalities, were incorporated at the C-10 position in genipin. Synthesized compounds showed weaker *ee*AChE inhibitory activity (less than 50% inhibition), but they were observed to be strong inhibitors of *eq*BuChE. In fact, the anti-BuChE activity of the majority of synthesized compounds was found to be greater than geniposide and genipin, suggesting that the incorporation of a 1,2,3-triazole ring augments the anti-ChE activity of genipin. Among all the synthesized compounds, acetoxy and hemiacetal 1,2,3-triazole genipin analogs, viz., 10-[4′-(7′-bromoethyl)-1*H*-1,2,3-triazole-1-yl]-1-acetoxygenipin (**5**; yield 75%) and 10-[4′-(6′,6′-diphenyl-6′-hydroxymethyl)-1*H*-1,2,3-triazole-1-yl] genipin (**6**; 64% yield), emerged as the most potent and selective BuChE inhibitors (IC_50_ = 31.8 and 54.3 μM, respectively). Compound **5**, with a bromoethyl–1,2,3-triazole scaffold, was found to be a better inhibitor than galantamine (IC_50_ = 34.1 μM), while **6**, containing a diphenylhydroxy group, displayed at par activity (IC_50_ = 54.3 μM) to a positive control (Figure 9). SAR studies of acetoxy genipin–1,2,3-triazole analogs revealed that the nature of the substituents at position 4 of the 1,2,3-triazole ring affect BuChE inhibition. The *para* substitution of a phenyl-1,2,3-triazole ring either with an electron-donating group (4-methoxy) or an electron-withdrawing group (4-fluoro) results in a decrease in activity. Similarly, replacing a phenyl ring of 1,2,3-triazole with di/triphenyl, benzyl ether, hydroxy-cyclic compounds, and benzylamine did not improve activity. However, 1,2,3-triazoles, upon replacing an aryl group with an alkyl chain, showed low-to-high inhibitory activity. In general, long-chain alkyl groups displayed lower inhibitory activity than the compound with a bromoethyl group (**5**). An improvement in activity was observed when long-chain alkyl groups are replaced with phthalimide moieties. Among the hemiacetal genipin–1,2,3-triazole derivatives, phenyl/diphenyl rings showed good-to-excellent inhibition against BuChE (67.51–99.85%). Inhibitory activity was found to decrease upon replacing the phenyl groups of **6** with phthalimide and hydroxy-cyclic moieties. Lineweaver−Burk plots of **5** and **6** confirmed them to be noncompetitive enzyme inhibitors with inhibition constants (KI, KIS), estimated to be 0.03 and 0.1 mM, respectively. Both the potent compounds protected the cells from H_2_O_2_-induced neurotoxicity. The BuChE inhibition mechanism of the most potent compounds, **5** and **6**, was studied with the help of molecular docking. Compounds **5** and **6** showed binding energies of −9.77 and −9.74 kcal/mol within the active site of BuChE (PDB code: 4BDS). The ester unit at the C4 position of the iridoid moiety in compound **5** and **6** formed three H- bonds with the His438 (catalytic subsite) and Ser198 of the CAS. The acetoxy carbonyl group also interacted with Trp82, Trp430, and Tyr440 residues to form three H- bonds. Both the acetoxy and ester unit interact in the CAS region to inhibit BuChE, while the 1,2,3-triazole ring interacts with Tyr332 and Asp70 in the PAS region via the H-bond and ionic interactions. On the other hand, the diphenylhydroxy groups of **6** are oriented toward the PAS region, forming two π–π interactions with Tyr332, while the iridoid fragment forms an H-bond with Trp82 residue in the CAS pocket.

It can be suggested that the introduction of substituted 1,2,3-triazoles to a genipin core structure improves their BuChE inhibitory potential, and, therefore, 1,2,3-triazole–genipin analogs such as **5** and **6** might serve as leading molecules in fighting AD [62].

### 3.3. Paeonol–1,2,3-triazole Hybrids

**Figure 10:**
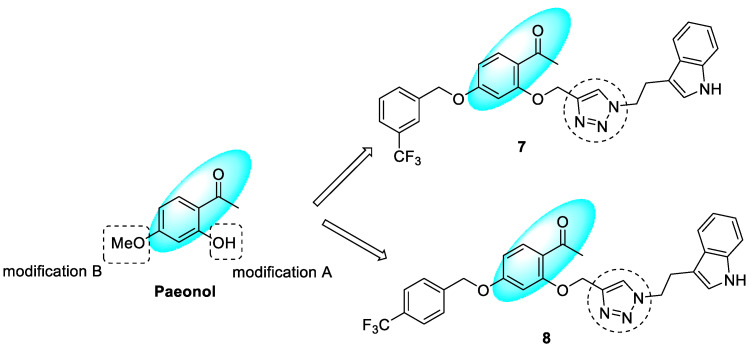
Chemical structures of paeonol–1,2,3-triazole hybrids.

It is well established that a low concentration of certain chemical neurotransmitters, including serotonin, epinephrine, norepinephrine, and dopamine, in the brain contributes to the development and progression of AD. The enzyme MAO-A is involved in the degradation of these neurotransmitters and causes catecholamine and 5-hydroxytryptamine (5-HT) inactivation, and, thus, MAO inhibitors can reduce the progression of AD. Paeonol is a phenolic derivative from the *Paeonia* genus herb with neuroprotective activity. 1,2,3-triazole linked with tryptamine-paeonol derivatives were synthesized and evaluated for AChE, BuChE, MAO-A, and MAO-B inhibitory activities. Compound **7** showed the most potent BuChE inhibition (IC_50_ = 0.13 µM) with a selectivity index of more than 769 for BuChE over AChE, whereas compound **8** showed selective MAO-B inhibition (Figure 10). In comparison with other derivatives, the *meta*-CF_3_ substituent increased the BuChE inhibitory activity, whereas the *para*-CF_3_ increased MAO-B inhibition. Compound **7** was found to be a reversible non-competitive BuChE inhibitor, whereas **8** was found to be a reversible competitive MAO-B inhibitor. Comparing the in-silico binding energies, compound **7** showed higher binding energy than **8** against BuChE (−13.75 vs. −11.29 kcal/mol); however, compound **8** has higher binding energy against MAO-B than MAO-A (−11.31 vs. −6.72 kcal/mol). The derivatives were slightly cytotoxic against normal cells (MDCK) and human neuroblastoma cells (SH-SY5Y) [63].

### 3.4. Quinazoline–1,2,3-triazole Hybrids

**Figure 11:**
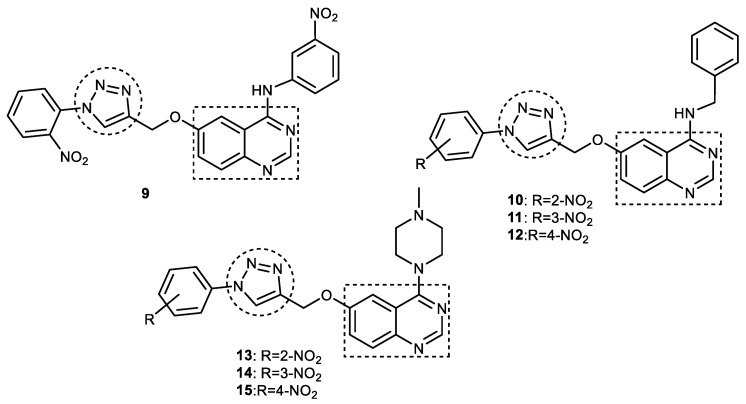
Chemical structures of quinazoline–1,2,3-triazole hybrids.

Quinazoline moiety is considered an important bioactive scaffold in designing therapeutic agents owing to its diverse array of biological properties [64,65]. This privileged structural motif has been shown to bind with both CAS and PAS on AChE, resulting in the significant inhibition of AChE [66]. Because of their powerful AChE inhibitory activities, quinazolines have been explored in designing anti-AD agents [66,67,68,69,70]. This was prompted by the results obtained by Rao and coworkers, who found that 2,4-disubstituted quinazolines bearing different primary amines at position C-4 of quinazoline exhibit promising anticholinesterase activities [70]. A 1,2,3-triazole nucleus containing a substituted aromatic ring at position 1 was linked through position C-6 to 4-amino-substituted quinazolines to obtain 4,6-disubstituted quinazoline–1,2,3-triazole hybrids, thus improving their AChE inhibitory activities. The 1,2,3-triazole nucleus was selected because it can form a hydrogen bond with the catalytic aspartate residue, and, secondly, various heterocyclic and or aromatic functionalities can be easily attached to it, helping in the optimization of physicochemical properties. The target quinazoline–1,2,3-triazole hybrids were prepared in a good yields (65–91%) by reacting intermediate 6-(prop-2-yn-1-yloxy) quinazolin-4-amines with different substituted arylazide derivatives using catalyst CuI in the presence of DIPEA in THF at an ambient temperature. All the twelve synthesized hybrid compounds inhibited the AChE enzyme (IC_50_ range = 0.2–83.9 μM), but it was observed that the inhibitory activity was influenced by the presence of the substituted amino group (3-nitrophenylamine < *N*-methylpiperazyl < benzylamine) at position C-4 of the quinazoline ring. Compound **10**, bearing a benzylamine moiety at the fourth position of the quinazoline ring and 2-nitrophenyl attached to a 1,2,3-triazole nucleus, was identified as the most potent AChE inhibitor (IC_50_ = 0.23 μM), though it was less active than donepezil (IC_50_ = 0.12 μM). The authors argued that benzylamine-containing hybrids are more flexible than other derivatives, which facilitates favorable binding to the ChE enzyme. It was observed that the presence of nitrophenyl (NO_2_-Ph) functionality in a 1,2,3-triazole ring imparts better inhibitory activity than the 3-trifluoromethyl-4-nitrile group. Furthermore, changing the position of the nitro group from *ortho* to *meta* in the phenyl ring resulted in a reduction in activity (1.10 μM), while the *para*-substituted compound was almost inactive (IC_50_ > 200 μM), suggesting that the electronic properties of substituents on the aromatic ring influence AChE inhibitory activity. Replacing the benzylamine group with the *N*-methylpiperazyl moiety on the C-4 position of quinazoline showed lower activity than corresponding compounds containing benzylamine, but, in contrast, *m*-nitrophenyl-1,2,3-triazole derivative **14** was found to be more active than *o*-nitrophenyl-1,2,3-triazole **13** by two times. *Para*-nitro-substituted compound **15** in the series displayed no activity, similar to **12**, suggesting that the 4-nitrophenyl group connected to the 1,2,3-triazole ring is detrimental to AChE inhibitory activity (Figure 11). SAR analysis pointed out that the nature of the amino group present at the C-4 position of the quinazoline moiety and nitrophenyl 1,2,3-triazole are crucial in imparting anti-AChE activities to hybrid molecules. Molecular docking studies suggest that compounds **9–11** are dual-binding site inhibitors of AChE, as they have been found to bind to both CAS and PAS at the active site of the AChE enzyme. Compound **9** forms interaction with Trp86 residue in the choline-binding region; its quinazoline ring binds to Trp286 via a π–π interaction and, similar to donepezil, forms hydrogen bonds with Phe295 and/or Ser293 residues. 1,2,3-triazole and nitrophenyl rings interact with Tyr341 and Try337 residues, respectively. Donepezil and compound **9** have been found to have similar docking scores, confirming their high affinity for ChE enzymes [71].

### 3.5. Quinoline-1,2,3-triazole Derivatives

**Figure 12:**
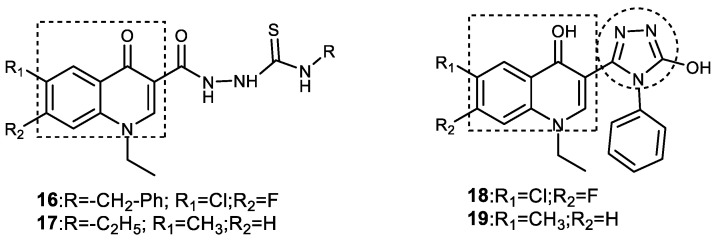
Chemical structures of quinoline–1,2,3-triazole conjugates.

Quinolone is a privileged, bicyclic, nitrogen-containing bioactive scaffold that is found in a large number of synthetic and natural products. It has been reported to exhibit a wide variety of biological and pharmacological activities, such as antiepileptic, antimalarial, antiviral, anticancer, antioxidant, etc. [72,73,74]. Quinoline-containing compounds are also used clinically to treat a variety of infectious diseases, including skin and soft-tissue infections [75,76]. Quinolines in the past were explored to design dual AChEIs, and, therefore, their role has been investigated in AD. Some of the quinolone-containing compounds that act at different molecular targets of AD include vitamin E or tocopherols (free radical scavengers), clioquinol (copper or iron chelator), and tacrine (AChEI). A series of new quinolone–1,2,3-triazole hybrid derivatives was designed, synthesized, and investigated for AChE inhibitory and antioxidant activity. All compounds except *N*-benzyl-2-(7-chloro-1-ethyl-6-fluoro-4-oxo-1,4-dihydroquinoline-3-carbonyl) hydrazine carbothioamide (**16**), 2-[(1-ethyl-6-methyl-4-oxo-1,4-dihydroquinolin-3-yl) carbonyl]-*N*-ethyl hydrazine carbothioamide (**17**), 7-chloro-1-ethyl-6-fluoro-3-(5-oxo-4-phenyl-4,5-dihydro-1*H*-1,2,4-triazol-3-yl) quinolin-4(1*H*)-one (**18**), and 1-ethyl-3-(5-hydroxy-4-phenyl-4*H*-1,2,4-triazol-3-yl)-6-methylquinolin-4(1*H*)-one (**19**) showed potent AChE inhibitory activities (IC_50_ = 0.48–0.64 mg/mL). The most potent compounds, **16–19**, showed IC_50_ values of 0.48, 0.52, 0.57, and 0.64 mg/mL in comparison with donepezil (IC_50_ = 0.03 mg/mL) (Figure 12). Molecular docking studies of **16** and **17** showed that the 1-ethylquinolin-4(1*H*)-one-3-yl moiety was oriented toward choline-binding pockets and engaged in strong π-π interactions with Trp286. They also form van der Waals interactions with Tyr72, Asp74, Tyr124, Tyr341, and Trp286 in the PAS region and with Trp86, Ser203, Tyr337, and Phe338 in the CAS region, similar to ligand–receptor interactions shown by anti-AD molecules such as tacrine, galantamaine, huperzine A, and donepezil. It is interesting to note that quinolone–carbothioamide compounds are more potent than quinolone-1,2,3-triazole hybrids [77].

#### Quinoline–1,2,3-triazole–Indanone Conjugates

**Figure 13:**
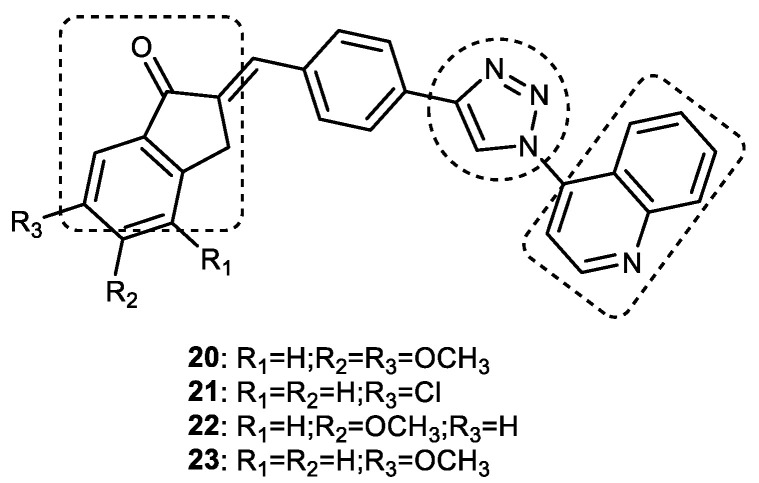
Chemical structures of quinoline-1,2,3-triazole-indanone trihybrids.

A library of new tacrine–donepezil rigid hybrids containing indanone-1,2,3-triazole-quinoline systems as potential dual-binding-site AChE inhibitors were synthesized by Mantoni et al. in 2016 [78]. Quinoline rings mimicking the acridine nucleus of tacrine and the dimethoxyindanone moiety from donepezil were linked through methine phenyl 1,2,3-triazole–quinoline systems to obtain a new structural scaffold to act as potent AChE inhibitors. Click chemistry was used to synthesize the target compounds. Briefly, 1,2,3-triazole-1,4-disubstituted alcohol in a 90% yield was prepared with a 1,3-dipolar cycloaddition reaction between 4-azidoquinoline and commercial 4-ethynylbenzyl alcohol using CuAAC under microwave irradiation for 10 min. This alcohol was oxidized for 5 min under microwave irradiation using pyridinium chlorochromate (PCC) to obtain a key aldehyde which was subjected to an aldol condensation reaction with indanone enolates followed by an E1cB elimination reaction to yield a series of conjugated 1,2,3-triazole–enone hybrids in good yield (75–96%). A few 1,2,3-triazole–quinoline conjugates (**20–23**) showed a 29–55% inhibition of *h*AChE at a concentration of 100 µM (Figure 13). (*E*)-5-methoxy-2-(4-(1-(quinolin-4-yl)-1*H*-1,2,3-triazol-4-yl) benzylidene)-2,3-dihydro-1*H*-inden-1-one (**22)** and (*E*)-6-methoxy-2-(4-(1-(quinolin-4-yl)-1*H*-1,2,3-triazol-4-yl) benzylidene)-2,3-dihydro-1*H*-inden-1-one (**23)** are the most potent and highly selective *h*AChE inhibitors, with IC_50_ values of 114 and 109 µM, respectively. The synthesized hybrid compounds were able to block both catalytic and peripheral AChE sites, making them an interesting structural scaffold for the development of potent AChE inhibitors. Similar to tacrine and donepezil, these compounds are also dual-binding-site (mixed-type) *h*AChE inhibitors [79,80].

A molecular docking analysis of compound **23** and donepezil on *h*AChE showed similar binding modes. Both formed interactions with the AChE catalytic triad residues Ser203, Glu334, and His447, as well as with Trp286 (π-π stacking with indanone) and Trp86 (π-π stacking with quinoline), located at the peripheral site and the catalytic cleft. The difference in activity between compound **23** and donepezil could be because of the formation of some additional interactions of **23** with Tyr124, Tyr337, and Tyr341 residues in the middle of the gorge. 1,2,3-triazole–quinoline scaffolds, because of their dual-binding mode to the active site of AChE, could be considered for further optimization to obtain potent ChE inhibitors [78].

### 3.6. Coumarin–1,2,3-triazole Hybrids

A coumarin scaffold is a privileged motif that, because of its potent anti-ChE activity, has been clubbed with various bioactive heterocyclic ring systems to obtain lead candidates. Coumarin–1,2,3-triazole hybrids from 2015–2020 have been reviewed [14]. The chemistry and neuroprotective actions of the most promising coumarin–1,2,3-triazole hybrids are presented in Table 2.

**Figure 14:**
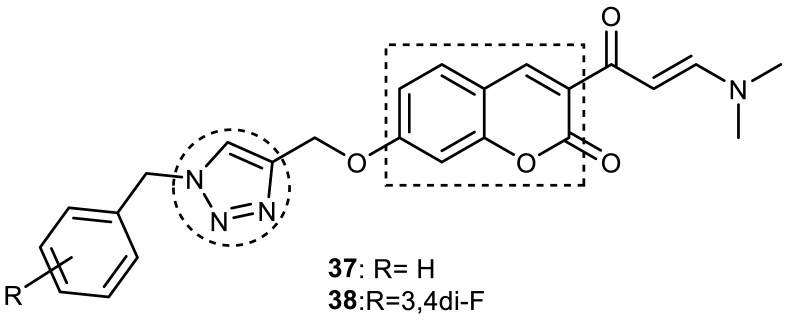
Chemical structure of coumarin–1,2,3-triazole hybrids.

Askarani et al., in 2020, used molecular hybridization and bioisosteric replacement strategies to synthesize 1,2,3-triazole-dimethylamino acryloyl-chromenones as MTDLs possessing anti-AChE/BuChE, anti-Aβ aggregation, neuroprotective, and metal-chelating properties beneficial in the treatment of AD. Thirteen new target compounds were prepared using a click reaction by reacting (*E*)-3-(3-(dimethylamino) acryloyl)-7-(prop-2-yn-1-yloxy)-2*H*-chromen-2-one and in situ-prepared aromatic azides in the presence of Et_3_N and CuSO_4_.5H_2_O in a mixture of water/*tert*-butanol at room temperature. Almost all of the synthesized compounds were inactive against AChE (IC_50_ => 100 μM), but, surprisingly, six out of thirteen 1,2,3-triazole–coumarin hybrids showed highly selective and potent anti-BuChE activity (IC_50_ = 21.71–65.96 μM); however, they were found to be slightly weaker than donepezil (IC_50_ = 5.19 μM). (*E*)-7-((1-(3,4-difluorobenzyl)-1*H*-1,2,3-triazol-4-yl) methoxy)-3-(3-(dimethylamino) acryloyl)-2*H*-chromen-2-one (**38**) was noted to be the most potent BuChE inhibitor (IC_50_ = 21.71 μM) (Figure 14). A SAR analysis indicated that the nature and position of the substituents on the benzyl ring in the 1,2,3-triazole nucleus influenced the BuChE inhibitory activity of the synthesized compounds. The unsubstituted benzyl derivative (**37**) moderately inhibited the BuChE activity (IC_50_ = 34.41 μM), but monosubstitution with an electron-donating group such as 3-OCH_3_ enhanced the BuChE inhibition (IC_50_ = 23.44 μM), while the addition of a weakly activating group (2-methyl) resulted in a decrease in activity (IC_50_ = 35.71 μM). Changing the position of a methyl group to the *para* position led to a loss of activity (IC_50_ > 100 μM). Placing a single halogen substituent at the *meta* position on the benzyl ring decreased the activity (F > Br > Cl; IC_50_ = 59.58 > 65.96 >> 100 μM), while the introduction of a halogen on either the *ortho* or *para* position abolished the BuChE inhibitory activity (IC_50_ => 100 μM). However, the addition of another fluorine atom, as in compound **38** (3,4-difluoro derivative), yielded the most potent BuChE inhibitor (IC_50_ = 21.71 μM). Based on the Lineweaver–Burk plot, **38** is classified as a mixed inhibitor with a calculated *Ki* value of 38.3 μM. Compound **38** was also found to inhibit self-induced Aβ_1–42_ aggregations (32.6%) and AChE-induced Aβ aggregation (29.4%), and it was successful in chelating biometals. It also exerted neuroprotective effects against Aβ_25–35_-induced PC12 cell injury. The results of molecular docking studies support the strong BuChE inhibitory activity of compound **38**. Two-dimensional interactions between compound **38** and BuChE showed that the carbonyl and oxygen of the pendant group in the chromenenone motif form an H-bond with Ser198 residue in the CAS region, and 3,4-difluoro benzyl fragment form π–π and van der Waals interactions with Tyr332 and target PAS pockets. In the best molecular docking pose, a 1,2,3-triazole ring interacted with the Trp82 of the anionic subsite via an H-bond and through van der Waals interactions with Ala326 residues in the PAS region. Another van der Waals interaction was observed between the nitrogen of dimethylamino propenone and Pro285 residue. The authors believed that dimethylamino propanone attached to the C-3 position in the chromenone ring and the nature/position of electronic substituents on the aryl ring attached to the 1,2,3-triazole ring are key structural features in designing potent BuChE inhibitors. However, it would be interesting to study the effect of replacing (i) the dimethylamino propenone pendant group with a small or bulky cyclic amine and (ii) using other linkers such as CO, NHCO, and NH in place of 1,2,3-triazoles on the potency and selectivity of BuChE inhibition. Thus, 1,2,3-triazole-dimethylamino acryloyl-chromenones synthesized using a rational hybridization approach could be taken up to further develop multifunctional candidates for AD therapy [93].

**Figure 15:**
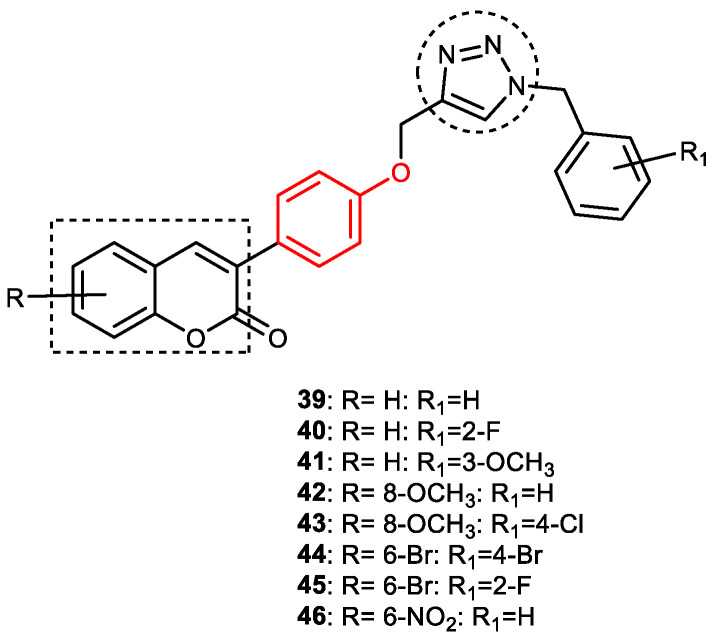
Chemical structure of coumarin–1,2,3-triazole hybrids.

Pourabdi et al., 2022, reported the synthesis, anti-ChE, 15-lipooxygenase (15-LOX), anti-Aβ aggregation, and neuroprotective activity of a novel series of 3-arylcoumarin derivatives linked to an *N*-benzyl 1,2,3-triazole ring system. They tried to obtain coumarin–1,2,3-triazole hybrids capable of exhibiting LOX inhibitory activity in addition to the ChE/neuroprotection/antioxidant activity. It was hypothesized that 3-arylcoumarins with suitable hydrophobic aryl rings are more likely to bind the LOX through hydrophobic interactions [94]. Hence, in an attempt to obtain MTDLs, their group conjugated two bioactive moieties into one structure, viz., 3-arylcoumarin linked with *N*-benzyl 1,2,3-triazole bearing some pharmacophoric groups known to augment/exhibit anti-ChE activity [95]. Hybrid compounds were synthesized in four steps. Azides for the final step were generated in situ from corresponding benzyl halides. A one-pot, three-component click chemistry reaction (CuAAC) was used to synthesize coumarin-linked 1,2,3-triazole hybrids. Synthesized hybrids displayed very weak anti-AChE activity; however, they were found to be weak to a moderate inhibitor of *eq*BuChE (IC_50_ = 6.7–36.5 μM) in comparison with tacrine (IC_50_ = 0.073 ± 0.009 μM). Among the unsubstituted/substituted coumarins, the 8-methoxy group substituted coumarin, namely, 3-(4-((1-(4-chlorobenzyl)-1*H*-1,2,3-triazol-4-yl) methoxy)phenyl)-8-methoxy-2*H*-chromen-2-one (**43**) and the 6-nitrocoumarin derivative 3-(4-((1-benzyl-1*H*-1,2,3-triazol-4-yl)methoxy) phenyl)-6-nitro-2*H*-chromen-2-one (**46**), showed the best anti-BuChE activity (IC_50_ = 6.7 and 6.3 μM, respectively). Unsubstituted coumarin derivatives (**39, 40**, and **41**) exhibited mild BuChE inhibitory activity (IC_50_ = 27.6, 36.5, and 19.5 μM, respectively). Surprisingly, 6-bromocoumarin derivatives (**44** and **45**) were inactive against BuChE (Figure 15). SAR studies also indicated that changing the type/position of the electronic parameters in the benzyl fragment contributes little to the BuChE inhibitory activity, while the type of substituent (6-NO_2_, 8-OCH_3_) on a coumarin ring can significantly influence the activity. Compound **41** (3-(4-((1-(3-methoxybenzyl)-1*H*-1,2,3-triazol-4-yl) methoxy) phenyl)-2*H*-chromen-2-one) and 3-(4-((1-benzyl-1*H*-1,2,3-triazol-4-yl)methoxy) phenyl)-8-methoxy-2*H*-chromen-2-one (**42**) exhibited acceptable 15-LOX inhibition activities (IC_50_ =39.1 and 41 μM, respectively) and were found to be 1.44- and 1.79-times better than donepezil in reducing the aggregation of Aβ_1–42_. Their neuroprotective effects against H_2_O_2_-induced toxicity were also stronger than the standard drug quercetin. Compound **41** also showed more activity than the positive antioxidant agent butylated hydroxytoluene (BHT). Molecular docking study results showed that the coumarin and 1,2,3-triazole rings of compounds **41** and **42** form different binding interactions with amino acid residues in the active site of BuChE. The *N*-benzyl 1,2,3-triazole of **41** bound to the catalytic triad, whereas the benzyl ring stacked with Trp82, and 1,2,3-triazole ring formed two T-shaped interactions with His438 and Trp82 residues. The coumarin ring was observed to form π stacking with Tyr332 and was leaning toward the rim of the BuChE. In the case of **42**, the coumarin moiety formed π stacking with Trp82 and lay at the bottom of the active site, while the *N*-benzyl 1,2,3-triazole tail was involved in H-bond formation with Asp70 at the gate of the active site [96].

#### 3.6.1. Coumarin–1,2,3-triazole–Tyrosol Trihybrids

**Figure 16:**
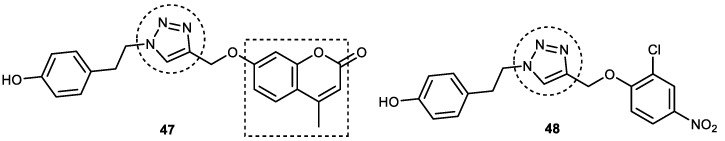
Chemical structure of coumarin–1,2,3-triazole–tyrosol trihybrids.

Tyrosol, chemically known as 2-(4-hydroxyphenyl) ethanol, is a major bioactive phenolic constituent found in wine and olive oil. Several studies have demonstrated that tyrosol contributes significantly to the useful antioxidant, anti-inflammatory, and neuroprotective effects of the Mediterranean diet [97,98]. Previous studies have also highlighted the potential of the Mediterranean diet in the prevention of AD [99,100]. Medicinal chemists often use the bioactive 1,2,3-triazole moiety to synthesize hybrid compounds by either attaching it to natural products/synthetic drugs or linking two or more scaffolds as the source of promising lead or therapeutic molecules [84,101,102]. Encouraged by the promising AChE inhibitory activity (67.8 % at 50 µmol/ L) of a coumarin–1,2,3-triazole-tyrosol hybrid, namely, 7-({1-[2-(4-hydroxyphenyl) ethyl]-1*H*-1,2,3-triazol-4-yl}methoxy)-4-methyl-2*H*-chromen-2-one (**47**), Bousada et al., in 2020, designed, synthesized, and evaluated tyrosol–1,2,3-triazole hybrids as a source of potent AChE inhibitors to treat AD. Click chemistry was used to synthesize the target hybrid compounds by reacting alkynes obtained via the propargylation of phenols, and the tyrosol-derived azide derivatives in good yields (27–98%). Tyrosol–1,2,3-triazole hybrids exhibited moderate-to-good anti-AChE activity (7.4–67.8% inhibition at 50 µM/L). The most active AChE inhibitor (**47**), which is a hybrid of tyrosol and coumarin connected through a 1,2,3-trizole spacer, showed more than 600% greater activity than the parent coumarin (IC_50_ = 14.66 µM), 4-methylumbelliferone. 4-(2-{4-[(2-chloro-4-nitrophenoxy) methyl]-1*H*-1,2,3-triazol-1-yl}ethyl)phenol (**48**), a hybrid of tyrosol and 1,2,3-triazole, showed a 55% inhibition of AChE at 50 µM (Figure 16). Kinetic studies showed it to be a competitive inhibitor of AChE. Further, a SAR analysis indicated that the substitution pattern of the 1,2,3-triazole ring and the tyrosol structural fragment plays an important role in AChE inhibition [103]. The results of the interaction affinity energy (**47** = −11.6 and **48** = −10.4 and tyrosol= −6.9 kcal/mol) were also in agreement with an in vitro AChE inhibitory assay.

#### 3.6.2. Coumarin–1,2,3-triazole–Amino Acid Trihybrids

**Figure 17:**
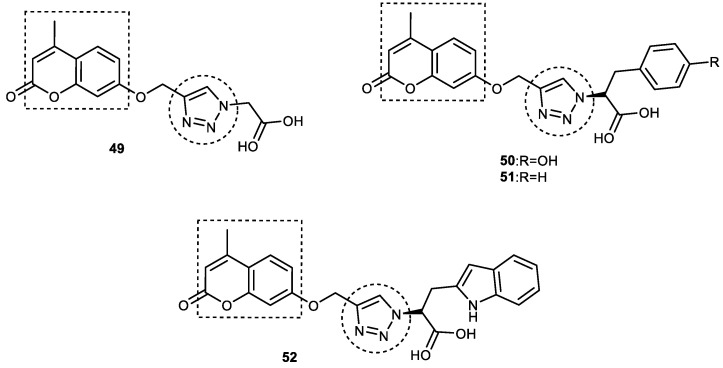
Chemical structure of coumarin–1,2,3-triazole–amino acid trihybrids.

In an attempt to improve the pharmacological and pharmacokinetic profile of **47**, Sousa et al., in 2021, prepared some coumarin–1,2,3-triazole–amino acid hybrids by condensing propargylated 4-methylumbelliferone with amino acid esters of glycine, tyrosine, tryptophan, and phenylalanine via a copper(I)-catalyzed azide alkyne cycloaddition reaction (CuAAC click chemistry) in moderate-to-good yields. They hypothesized that aliphatic amino acid hybrids would exhibit weaker anti-AChE activity in comparison with aromatic amino acid derivatives. The esterification of amino acids was carried out to improve the solubility of hybrids in organic solvents. 1,2,3-triazole hybrids were then further hydrolyzed to yield coumarin–amino acids connected through a 1,2,3-triazole moiety. The anti-AChE activity of the coumarin–1,2,3-triazole–amino acid hybrids (**49–52**) and compound **47** were tested at concentrations of 50, 100, and 200 μM (Figure 17). The results of the AChE inhibition assay showed a dose-dependent increase in inhibitory activity in the order of t-Tryp-COOH (**52**) > t-Phe-COOH (**51**) > t-Tyr-COOH (**50**) > t-Gly-COOH (**49**). It is evident that aromatic amino acid-derived hybrids are more powerful inhibitors than glycine (an aliphatic amino acid) derivatives at all concentrations. At a 200 μM concentration, compound **49**, a 1,2,3-triazole–GlyCOOH hybrid, inhibited only 15.84 ± 1.51% of AChE activity, while compound **52** (t-Tryp-COOH) showed 43.26% in comparison with compound **47** (72.38%) and a galantamine positive control (89.2% inhibition at 17 µM). In general, tryptophan and phenylalanine-1,2,3-triazole hybrids were found to be more promising than tyrosine. The findings of this study indicated that replacing tyrosol with tyrosine, tryptophan, phenylalanine, and glycine amino acids in the 1,2,3-triazole ring does not improve the anti-AChE spectrum. Conversely, the presence of a carboxylic acid group in 1,2,3-triazole hybrids decreases AChE inhibitory activity [104].

### 3.7. Tacrine–1,2,3-triazole Hybrids

**Figure 18:**
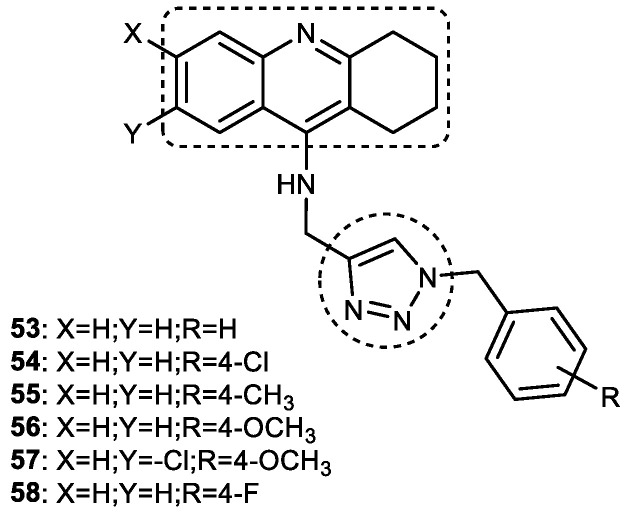
Chemical structure of tacrine–1,2,3-triazole hybrids.

The synthesis and anti-ChE activity of fifteen new tacrine–1,2,3-triazole hybrids were reported by Najafi et al. in 2016. Acridine derivatives were propargylated, which were then subjected to a click reaction with in situ-prepared organic azide derivatives to obtain tacrine–1,2,3-triazole hybrids in a moderate-to-good percentage yields. Nearly all the hybrid molecules showed promising dual ChE inhibition (IC_50_ for AChE = 0.521–3.0 μM and 0.059->5 μM against BuChE). The AChE and BuChE inhibitory activities of the tacrine–1,2,3-triazole hybrids were 3-21- and 2-140-fold more powerful than the standard drug rivastigmine (IC_50_ for AChE = 11.07 μM, and 7.72 μM against BuChE) but were much weaker than tacrine (IC_50_ for AChE = 0.048 μM, and 0.0.01 μM against BuChE). Among all the hybrids, 7-chloro-*N*-((1-(4-methoxybenzyl)-1*H*-1,2,3-triazol-4-yl)methyl)-1,2,3,4-tetrahydroacridin-9-amine (**57**) and *N*-((1-(4-methoxybenzyl)-1*H*-1,2,3-triazol-4-yl)methyl)-1,2,3,4-tetrahydroacridin-9-amine (**56**) demonstrated the most potent and highly selective anti-AChE (IC_50_ = 0.521 μM; SI for AChE = 3.55) and anti-BuChE activities (IC_50_ = 0.055 μM; SI for BuChE = 36.36), respectively (Figure 18). SAR studies showed that replacing 4-OCH_3_ in the benzyl ring attached to 1,2,3-triazole with methyl, chlorine, fluorine, and hydrogen resulted in a decrease in AChE inhibition. Further, changing the position of 7-Cl to 6-Cl in the acridine ring of **57** led to a slight reduction in the AChE inhibitory activity. It was also observed that the AChEI activity of the hybrids was affected by the electronic character of the substituents on the benzyl ring connected to the 1,2,3-triazole and acridine rings because the removal of all the substituents, i.e., the unsubstituted acridine moiety and benzyl ring (**53**), exhibited the lowest AChE inhibition among all the synthesized compounds. Therefore, it can be inferred that the presence of a *para* substituent, preferably CH_3_ > Cl > OCH_3_ > F > H in the benzyl ring, improves the activity (**55 > 54 > 56 > 58 > 53**). However, most potent AChE inhibitors possess two substituents, one in each acridine and aryl ring connected to 1,2,3-triazole. Two hybrids bearing no substituents in acridine moiety (**56**) and *N*-((1-(4-fluorobenzyl)-1*H*-1,2,3-triazol-4-yl) methyl)-1,2,3,4-tetrahydroacridin-9-amine (**58**), displayed the best BuChE inhibitory activities (IC_50_ = 0.055 and 0.059 μM, respectively). Interestingly, changing the position of a 6-chloro substituent to the seventh position on the acridine ring resulted in a decrease in BuChE inhibition. Hybrids in which the benzyl ring was mono-substituted and the acridine possessed no substituents showed the lowest selectivity toward BuChE inhibition. In addition, a reduction in BuChE inhibition was noted with the substituents in the following order: OCH_3_ > F > CH_3_ > Cl. In kinetic studies, compounds **57** and **56** showed mixed inhibition with K*i* values of 0.94 μM and 0.121 μM, respectively. They were found to bind simultaneously to the PAS and catalytic sites of ChE. Compound **57** interacted with the Trp279 and Tyr121 residues of AChE by forming two H-bonds through the methoxy group and the 1,2,3-triazole ring. The aromatic ring of 4-methoxyphenyl interacted with Tyr334 via a π-π interaction, while acridine formed π-π stacking with the Trp84 and Phe330 of CAS. Chlorine present on the acridine ring showed a hydrophobic interaction with Tyr334, Phe330, and Trp432. Additionally, in vitro and in vivo evaluations of the most potent AChEI (**57**) showed moderate neuroprotective activity and memory improvement in scopolamine-induced memory impairment. In general, these compounds appear to be dual inhibitors but are more selective toward BuChE and have therapeutic potential for the treatment of AD [105].

#### 3.7.1. Tacrine–1,2,3-triazole–Quinoline Trihybrids

**Figure 19:**
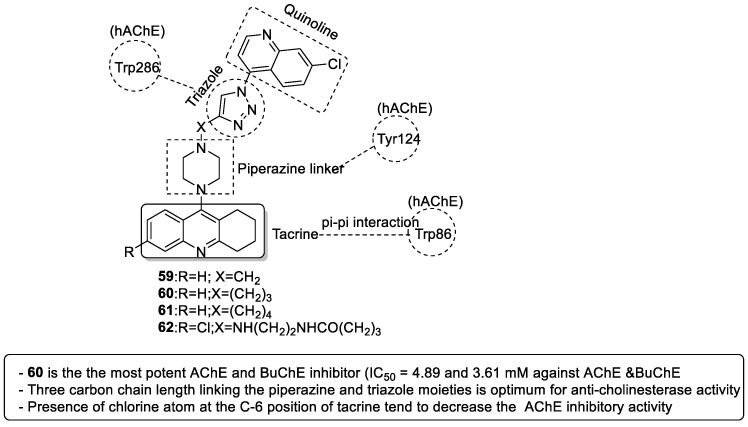
Chemical structure of tacrine–1,2,3-triazole–quinoline trihybrids.

Wu et al. (2018) synthesized 11 new tacrine–1,2,3-triazole–quinoline hybrids and tested their anti-ChE activity against *ee*AChE and *horse serum* BuChE enzymes to develop anti-AD drugs. The target compounds were obtained in moderate-to-high yields by reacting 4-azido-7-chloroquinoline and 9-(4-alkyne substituted piperazin-1-yl)-1,2,3,4-tetrahydroacridine intermediates in the presence of triethylamine and sodium ascorbate in *n*-butanol/water (1:1 v/v) via a Cu(I))-catalyzed alkyne–azide 1,3-dipolar cycloaddition (CuAAC click chemistry) reaction. Five out of eleven synthesized compounds were able to inhibit more than 60% of AChE activity at 100 μM. However, 9-(4-(3-(1-(7-chloroquinolin-4-yl)-1*H*-1,2,3-triazol-4-yl)-propyl) piperazin-1-yl)-1,2,3,4-tetrahydroacridine (**60**) was identified as the most potent AChE and BuChE inhibitor, exhibiting inhibitory activities of 78.7% and 91.8% at 100 μM (IC_50_ = 4.89 and 3.61 μM, respectively) (Figure 19). Although compound **62** showed a two times higher selectivity for BuChE (82.7% inhibition) over AChE (49.4% inhibition), its IC_50_ value for BuChE (6.06 μM) was much lesser than compound **60**. Overall, tacrine–1,2,3-triazole–quinoline hybrids were found to be weaker ChE inhibitors than tacrine (IC_50_ = 0.316 and 0.066 μM for AChE and BuChE; SI = 4.78). SAR studies indicated the following: (i) A three-carbon chain length linking the piperazine and 1,2,3-triazole moieties is optimal for anti-ChE activity (**60**). A decrease in ChE inhibitory activity would result if the linker length was either increased or shortened (**60 > 61 > 59**). (ii) The presence of a chlorine atom at the C-6 position of tacrine was also observed to decrease the inhibitory activity. Molecular docking studies revealed a similar binding interaction mode for **60** in the active sites of *h*AChE (PDB ID: 1B41) and *h*BuChE (1P0I), viz., the acridine ring system interacted with Trp86 (*h*AChE) and Trp82 (*h*BuChE) through a π–π interaction in the CAS region, while piperazine and 1,2,3-triazole interacted with Tyr124 and Trp286 residues in *h*AChE. In the PAS region of *h*BuChE, substituted quinolines present at the C-1 position of the 1,2,3-triazole ring were found to form π–π interactions with Ser287 and Pro285 residues. The authors suggested that the weaker anti-ChE activity of **60**, in comparison with tacrine, could be due to the rigidity of the 1,2,3-triazole and piperazine rings in addition to the presence of many polar groups in its structure, which unfavorably contributes to the hydrophobicity of the active sites in the PAS region [106].

#### 3.7.2. Tacrine–1,2,3-triazole–chalcone Hybrids

**Figure 20:**
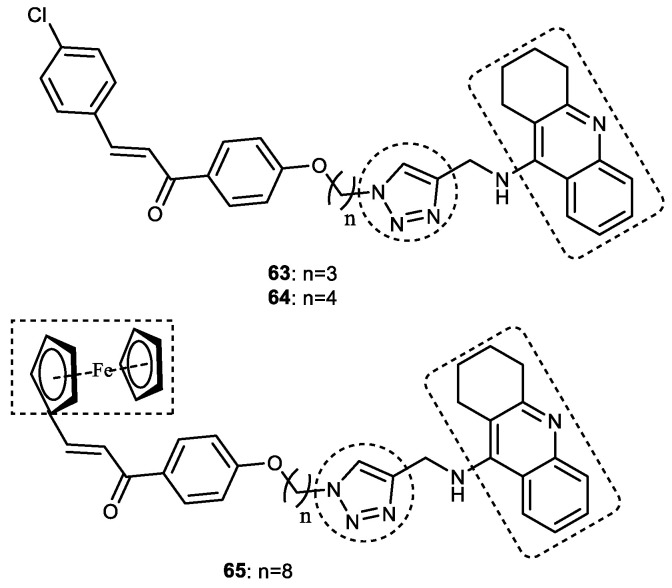
Chemical structure of tacrine–1,2,3-triazole–chalcone trihybrids.

Tacrine is a low-molecular-weight compound and is an excellent ChE inhibitor. Despite its hepatotoxicity, tacrine is still used by medicinal chemists to develop MTDLs for AD [25,26]. Several previous studies tried to reduce its hepatotoxicity by conjugating or coupling the tacrine motif with antioxidant and hepatoprotective molecules [107]. Chalcones or benzyl acetophenones, or α, β-unsaturated ketones (1,3-diaryl-2-propen-1-ones), are part of natural products such as curcumin, butein, cardamonin, and isoliquiritigenin. Chalcones, owing to their useful anti-AChE, neuroprotective, antioxidant, anti-inflammatory, and vasodilator activities [108], are considered a versatile motif by medicinal chemists. This prompted Rani et al. to synthesize various tacrine–chalcone analogs linked via 1*H*-1,2,3-triazole. They reacted *O*-alkyl azido chalcones/*O*-alkylazido ferrocenyl chalcone with *N*-alkylated tacrine using a Cu-promoted azide–alkyne cycloaddition reaction to obtain 1*H*-1,2,3-triazole tethered tacrine–chalcone conjugates as a source of AChE and BuChE inhibitors. An in vitro AChE inhibition assay revealed that three compounds—(*E*)-3-(4-chlorophenyl)-1-(4-(3-(4-(((1,2,3,4-tetrahydroacridin-9-yl)amino)methyl)-1*H*-1,2,3-triazol-1-yl)propoxy)phenyl)prop-2-en-1-one **63** (IC_50_ = 0.259 μM), (*E*)-3-(4-chlorophenyl)-1-(4-(4-(4-(((1,2,3,4-tetrahydroacridin-9-yl)amino)methyl)-1*H*-1,2,3-triazol-1-yl)butoxy)phenyl) prop-2-en-1-one **64** (IC_50_ = 0.372 μM), and (*E*)-3-(ferrocenyl)-1-(4-((8-(4-(((1,2,3,4-tetrahydroacridin-9-yl)amino)methyl)-1*H*-1,2,3-triazol-1-yl)octyl)oxy)phenyl)prop-2-en-1-one **65** (IC_50_ = 0.327 μM)— were more potent than the standard drug tacrine (IC_50_ = 0.375 μM). These compounds did not show any acute toxicity and, thus, were further tested for anti-BuChE activity. A SAR analysis revealed that the nature of the substituents on the A ring of the chalcone, as well as the carbon chain length of the spacer between the chalcone and 1,2,3-triazole, influenced the AChE inhibitory activity. Conjugates having a 4-Cl group on ring A of the chalcone showed higher activity than other substituents (4-F, 4-OCH_3_, 2,3,4 tri -OCH_3_). The optimal chain alkyl chain length was noted to be *n* = 3 or 4 because increasing the chain length resulted in a reduction in AChE inhibition. All five ferrocenyl conjugates showed lower activity than compound **63**, and only **65** (*n* = 6) displayed at par AChE inhibition (IC_50_ = 0.375 μM) to that of tacrine (Figure 20). Furthermore, only compound **64** showed promising activity against BuChE (IC_50_ = 5.328 μM), suggesting that these hybrid molecules are selective AChE inhibitors. The results of in vivo studies demonstrated that compounds **63** and **64** could reverse scopolamine-induced oxidative stress. In molecular docking studies performed using AChE (PDB id: 1H22), a docking score of **63** (−14.189 kcal/mol) was better than **64** (−13.459 kcal/mol), which clearly supported the results of the in vitro experiments. It was observed that the π–π, π-cationic, and salt bridge interactions of **63** influenced its binding mode with AChE. The benzene ring in the ethoxy benzene fragment, benzene ring, and pyridine ring of the tacrine of **63** showed π–π interactions with Tyr334 and Phe330, respectively. Two π-cationic interactions were seen between the NH group of the pyridine ring of **63** and Phe330 and Trp84 residues. The NH group of the pyridine ring also formed a salt bridge interaction with the carboxylate group of Asp72. Compound **64** showed a slightly different binding affinity than **63** and was involved in one H-bond (the C=O of the benzophenone and Ser286) and two π-cationic interactions between the tacrine moiety and Trp84. Both **63** and **64** fit snugly in the active site cavity of AChE, which is mostly forming hydrophobic interactions. Based on in vitro and molecular docking studies, it can be suggested that 1*H*-1,2,3-triazole-tethered tacrine–chalcone conjugates may prove to be a valuable scaffold for the treatment of neurodegenerative diseases such as AD [109].

#### 3.7.3. Tacrine–1,2,3-triazole–coumarin trihybrids

**Figure 21:**
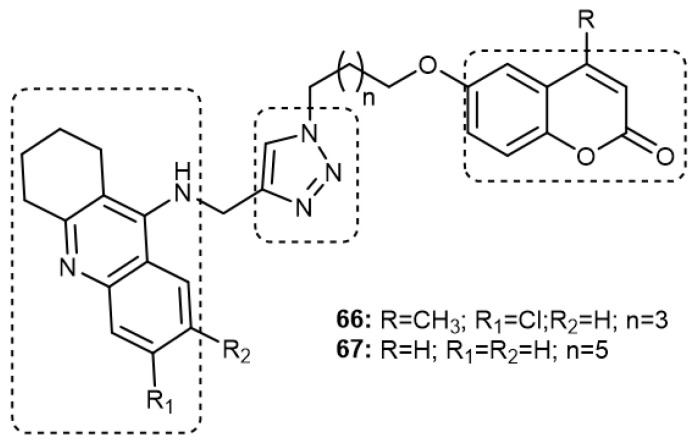
Chemical structure of tacrine–1,2,3-triazole–coumarin trihybrids.

Najafi et al., in 2019, prepared tacrine–1,2,3-triazole–coumarin trihybrids and tested their anti-Alzheimer’s activity through various bioassays and in silico studies. Compounds **66** and **67** were identified as the most potent AChE and BuChE inhibitors (IC_50_ values of 27 nM and 6 nM, respectively) (Figure 21). The SAR studies indicated that the presence of 4-CH_3_ group in the coumarin ring and the -Cl group in the tacrine fragment increased AChE inhibitory activity. The length of the methylene linker also affects the anti-AChE activity because of its different lipophilicity and spatial hindrance [110].

#### 3.7.4. Tacrine–1,2,3-triazole Glycoconjugates

**Figure 22:**
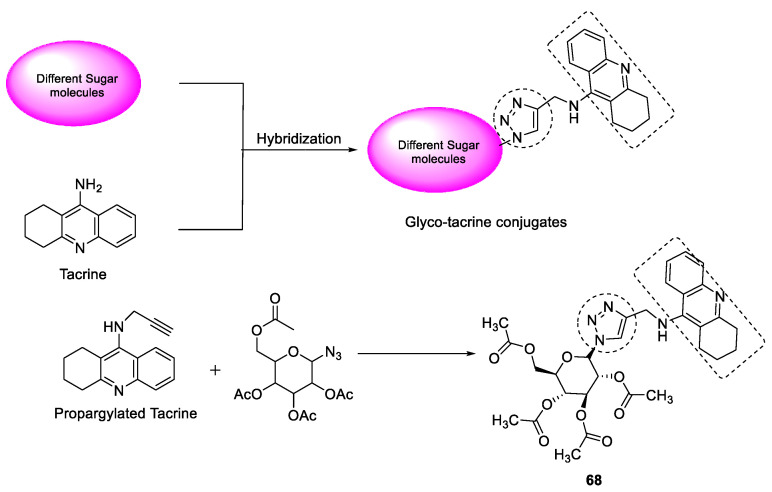
Chemical structure of tacrine–1,2,3-triazole glycoconjugate.

Some new tacrine-linked 1,2,3-triazole glycoconjugates were synthesized using Huisgen’s [3 + 2] cycloaddition between anomeric azides and tacrine-containing terminal acetylenes. Because the drug tacrine is associated with hepatotoxicity, therefore, the compounds were evaluated for hepatotoxicity studies along with in vitro AChE inhibitory activity. The compounds were found to be nontoxic to HePG2 cell lines at 200 µM after 24 h of incubation. Compound **68** showed the most potent AChE inhibitory activity (Figure 22) at IC_50_ of 0.4 µM and may be considered a versatiletemplate for the further development of drugs for AD [111].

### 3.8. Metalophthalcyanines–1,2,3-triazole Hybrids

**Figure 23:**
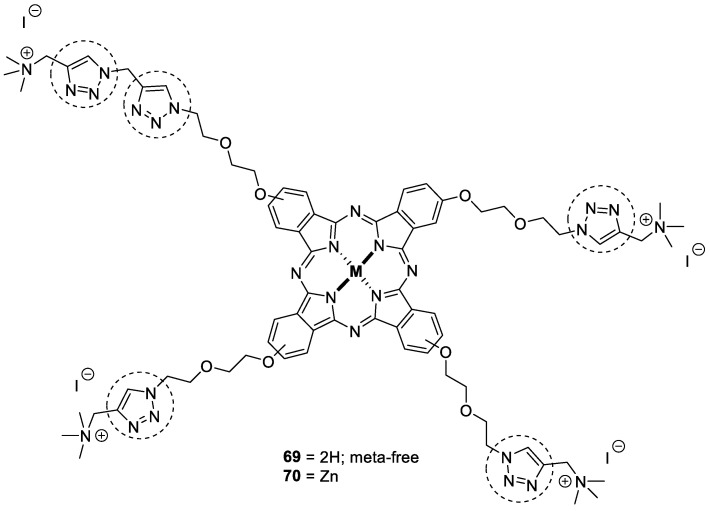
Chemical structure of metalophthalocyanines–1,2,3-triazole conjugates.

For the first time, metalophthalocyanines substituted with 1,2,3-triazole and their water-soluble derivatives were synthesized and tested for AChE and BuChE inhibition. The water-soluble derivatives were more potent, and among them, compound **69** showed selectivity against AChE in s micromolar range (IC_50_ = 0.040 µM), while compound **70** was a potent inhibitor of BuChE with an IC_50_ value of 0.1198 µM (Figure 23) [112].

**Figure 24:**
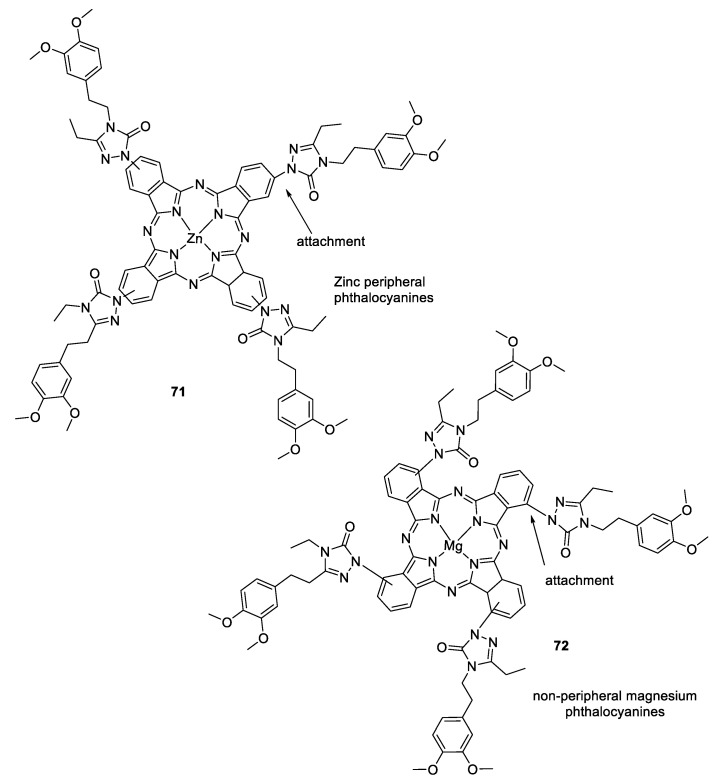
Chemical structures of metalophthalocyanines–1,2,3-triazole conjugates.

1,2,3-triazole derivatives substituted with zinc, magnesium, and lead phthalocyanines have also been synthesized. The synthesized derivatives showed outstanding solubility in most of the common organic solvents, including 1,4-dioxane, chloroform, acetonitrile, DMF, DMSO, tetrahydrofuran, and ethyl acetate. Peripherally tetra-substituted zinc phthalocyanine (**71**) generates high singlet oxygen in DMSO as compared with unsubstituted zinc phthalocyanine; thus, it may be explored further for photosensitizers in photodynamic therapy. The effective generation of single oxygen acts as an effective photosensitizer in photodynamic cancer treatment. The nonperipheral magnesium phthalocyanine (**72)** has shown the highest inhibitory activity against AChE (IC_50_ = 37.98 µM) and α-glucosidase (IC_50_ = 54.05 µM) using spectrophotometric methods (Figure 24) [113].

### 3.9. Chalcone–1,2,3-triazole Hybrids

**Figure 25:**
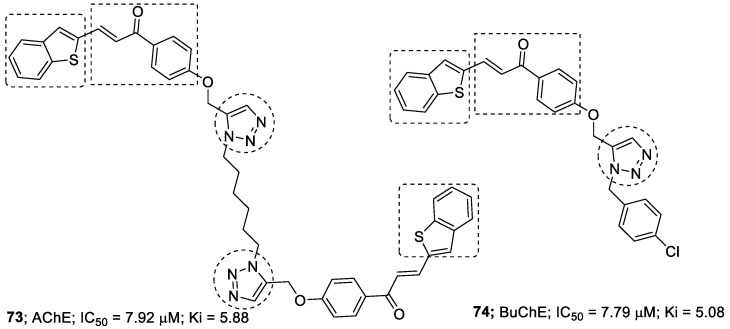
Chemical structures of chalcone–1,2,3-triazole hybrids.

A chalcone core with 1,2,3-triazole derivatives were synthesized, characterized and evaluated for their inhibitory activity against AChE and BuChE. The synthesized compounds showed good inhibitory activity against two ChE enzymes, AChE and BuChE, with K*i* values in the range of 5.88–11.13 µM and 5.08–15.12 µM. The derivatives with benzothiophene showed the most potent inhibitory activities against AChE (IC_50_ = 7.92 µM) and BuChE (IC_50_ = 7.79 µM). The best inhibitory activities against AChE and BuChE were shown by compound **73** and compound **74** (Figure 25) [114].

### 3.10. Benzimidazole–1,2,3-triazole Hybrids

**Figure 26:**
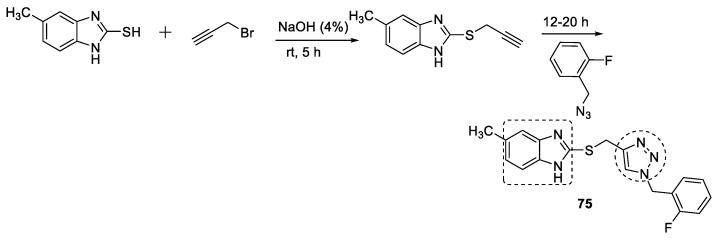
Chemical structure of benzimidazole–1,2,3-triazole hybrid.

Benzfused heterocycles, i.e., benzimidazole linked with 1,2,3-triazole, were synthesized and evaluated for in vitro AChE inhibitory activity. The compounds showed AChE inhibition, and the most active hybrid among them was 1-(2-fluorobenzyl)-1,2,3-triazole linked to benzimidazole ring (**75**), which showed 84% inhibition at 100 µM. The synthesis was carried out through a click reaction between the benzyl azide and 2-(prop-2-yn-1-ylthio)-1*H*-benzo[*d*]imidazole (Figure 26). The docking simulation confirmed that the compounds bound selectively to the cationic site of the enzyme [115].

### 3.11. Carbazole–1,2,3-triazole Hybrids

**Figure 27:**
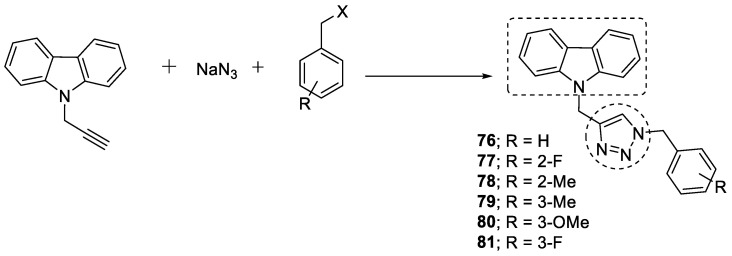
Chemical structures of carbazole-1,2,3-triazole hybrids.

A new series of anti-AChE compounds containing 1,2,3-triazole-linked carbazole was designed and synthesized. The synthesis was performed via one-pot three component click chemistry approach using *N*-propargyl-9*H*-carbazole, sodium azide, and an appropriate benzyl halide via copper-catalyzed cycloaddition. The unsubstituted benzyl (**76**) and the ring-substituted compound with 2-F (**77**), 2-CH_3_ (**78**), 3-CH_3_ (**79**), 3-OCH_3_ (**80**), and 3-F (**81**) possessed significant AChE activity (IC_50_ range ≤ 3.8 µM) (Figure 27). The compound with 2-CH_3_ was the most potent compound, with an IC_50_ value of 1.9 µM against AChE. It was further found that small halogen groups, such as fluorine, and electron-donating groups, such as methyl and methoxy, at the *ortho* or *meta* positions of the benzyl showed improvement in AChE inhibitory activity [116].

### 3.12. 1,4-Naphthoquinone–1,2,3-triazole Hybrids

**Figure 28:**
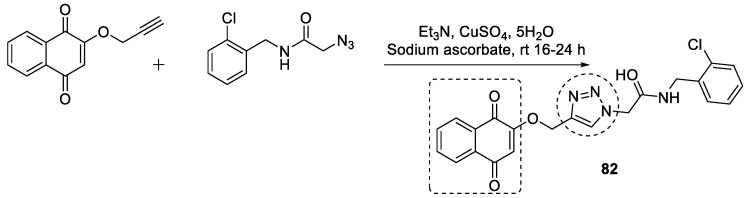
Chemical structure of 1,4 naphthoquinone–1,2,3-triazole hybrid.

The potential target for AD, i.e., AChE and BuChE, led Hosseini et al. to design and synthesize 1,4-naphthoquinone with 1,2,3-triazole acetamide derivatives. The anti-ChE activity and molecular docking confirmed that compound **82** possessed the strongest inhibition against both AChE and BuChE, with K*i* values of 10.16 and 8.04 nM, taking tacrine as a positive control with K*i* = 70.61 and 64.18 nM (Figure 28). Compound **82** binds to both the active sites of AChE and BuChE [117].

### 3.13. Oxadiazole–1,2,3-triazole Hybrids

**Figure 29:**
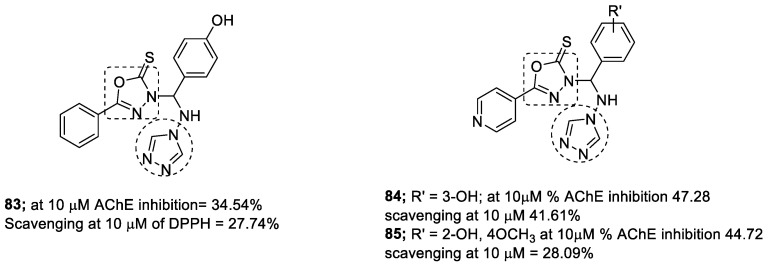
Chemical structures of oxadiazole–1,2,3-triazole hybrids.

1,2,3-triazole-oxadiazole conjugates have been designed, synthesized, and evaluated against in vitro AChE. The level of oxidative stress biochemical markers, such as lipid peroxidation, superoxide dismutase, glutathione, and catalase induced by the scopolamine, were also evaluated in the presence of the compounds. Among them, derivatives **83–85** showed good activity against AChE (34.54–47.28% inhibition at 10 µM) (Figure 29). The compounds also showed a promising role in decreasing oxidative stress (27.74–41.61% inhibition of DPPH at 10 µM). In molecular docking studies against recombinant *h*AChE, the compounds showed binding with amino acid residues at the active site of the receptors [118].

### 3.14. Acridone–1,2,3-triazole Hybrids

**Figure 30:**
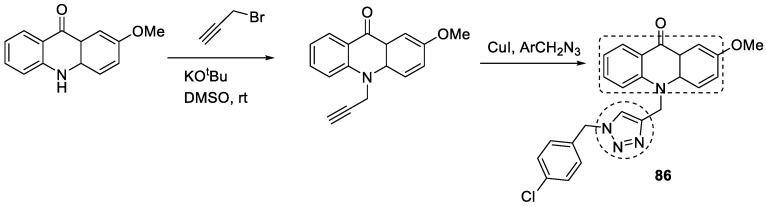
Chemical structure of acridone–1,2,3-triazole hybrid.

Khanaposhtani et al. linked 1,2,3-triazole with acridone to obtain compounds with AChE and BuChE inhibitory activities. The acridone formation takes place with the cyclization reaction of 2-bromobenzoic acid and aniline derivatives, then acridone reacts with propargyl bromide via a click reaction by azide–alkyne cycloaddition to obtain the final compounds. Among the synthesized compounds, **86**, i.e., 10-((1-(4-chlorobenzyl)-1*H*-1,2,3-triazol-4-yl) methyl)-2-methoxyacridin-9(10*H*)-one, showed the most potent AChE inhibitory activity, with an IC_50_ value of 7.31 µM (Figure 30) [119].

### 3.15. Donepezil–1,2,3-triazole Hybrids

**Figure 31:**
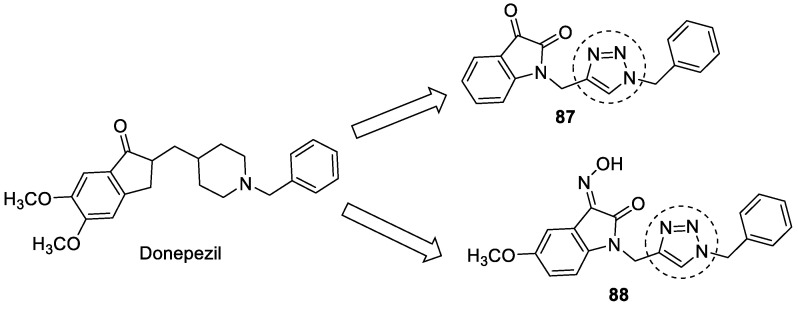
Chemical structure of donepezil–1,2,3-triazole hybrid.

Donepezil, a well-known AChE inhibitor, was used to design a series of indoline-2-one derivatives with 1-benzyl-1*H*-1,2,3-triazole moieties as potent AChE inhibitors. The two compounds **87** and **88** were found to be the most potent AChE inhibitors, with 51 and 50% inhibition at a concentration of 100 µM (Figure 31) [120].

**Figure 32:**
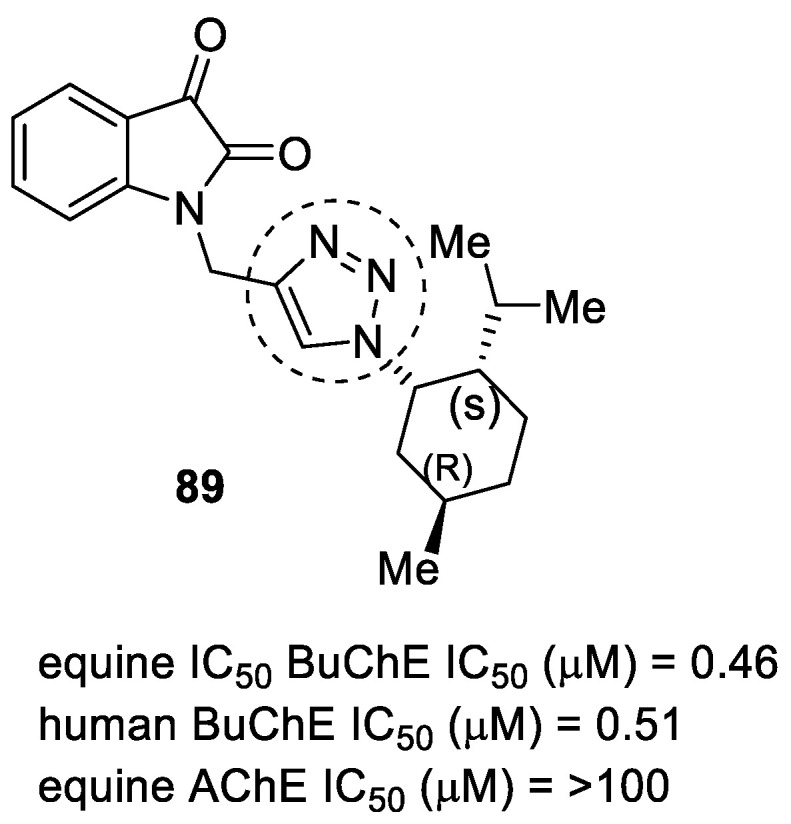
Chemical structures of donepezil–1,2,3-triazole hybrids.

Several istain-*N*-1,2,3-triazole derivatives were synthesized to target Aβ and ChE enzymes. Compound **89** showed more activity against *equine* and *human* BuChE enzymes (Figure 32). The best inhibition was against *equine* BuChE, with an IC_50_ of 0.46 µM. The compounds were non-hepatotoxic and weakly neurotoxic, and they were evaluated using neurite outgrowth experiments. The compound demonstrated a weak anti-aggregation effect [121].

**Figure 33:**
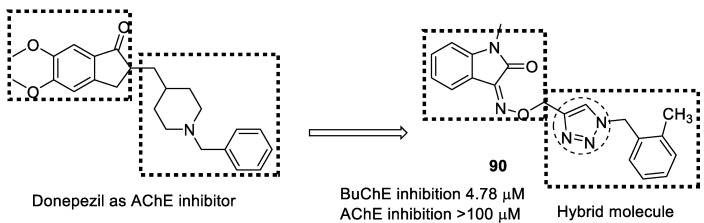
Chemical structure of donepezil–1,2,3-triazole hybrid.

The linker benzyl-1,2,3-triazole was used for molecular hybridization with methyl indolinone, as benzyl-1,2,3-triazole possesses a high dipole moment and is capable of forming hydrogen bond interactions with the active sites. The synthesized compound showed good-to-moderate activity against BuChE but weak AChE inhibition. Compound **90** was found to be the most potent, with an IC_50_ value of 4.78 µM, and is more potent than the standard donepezil (5.19 µM) (Figure 33). Docking studies confirmed that compound **90** binds to both the PAS and CAS sites of the BuChE. The compound protected and prevented the death of the PC12 neuronal cell line against toxicity induced by Aβ_25–35_, which was assayed using an MTT assay and did not show toxicity up to a conc. of 50 µM; however, it was devoid of β-site APP-cleaving enzyme-1 (BACE1) inhibitory activity in a dose range of 10–50 µM [122].

**Figure 34:**
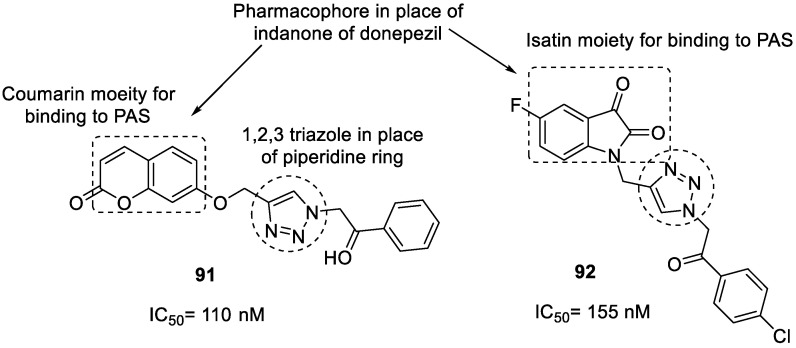
Chemical structure of donepezil (coumarin/isatin)–1,2,3-triazole hybrids.

Bhagat et al., in 2021, used a rational drug design approach to synthesize two series of donepezil-based hybrid molecules using click chemistry following copper-catalyzed 1,4 cycloaddition reactions. The indanone moiety of donepezil was replaced with either coumarin or isatin scaffolds to improve its aromatic binding to PAS on AChE while, to further improve interactions with CAS, a 1,2,3-triazole moiety-bearing benzoyl group was incorporated in place of the piperidine ring and benzyl fragment. Compounds in both series showed significant in vitro inhibitory activity against *eel*AChE; however, coumarin–1,2,3-triazole hybrids were noted to be more powerful inhibitors (IC_50_ = 110–502 nM; 12.85–87.14% inhibition at 1 µM) than isatin–1,2,3-triazole hybrids (IC_50_ = 155–852 nM; 28.57–96.58% inhibition at 1 µM). Compounds **91** (4-((1-(2-oxo-2-phenylethyl)-1*H*-1,2,3-triazol-4-yl)methoxy)-2*H*-chromen-2-one) and **92** (1-((1-(2-(4-chlorophenyl)-2-oxoethyl)-1*H*-1,2,3- triazol-4-yl)methyl)-5-fluoroindoline-2,3–dione), the two most potent AChEIs (IC_50_ = 110 and 155 nM, respectively), were found to be mixed inhibitors of AChE, and they preferentially bind to the free enzyme instead of the enzyme–substrate complex (Figure 34). These compounds showed similar binding interactions to donepezil and were stable in the active site, as revealed by molecular dynamic simulations. However, their inhibitory activity was slightly weaker than the parent drug donepezil (IC_50_ = 42 nM; 99.01 % inhibition at 1 µM). SAR studies revealed that the presence of an electronegative group such as F, Cl, or NO_2_ on the benzoyl ring improves AChE inhibition. These two hit/lead molecules may be further optimized to obtain potent AChEIs [123].

### 3.16. Benzodiazepine–1,2,3-triazole Hybrids

**Figure 35:**
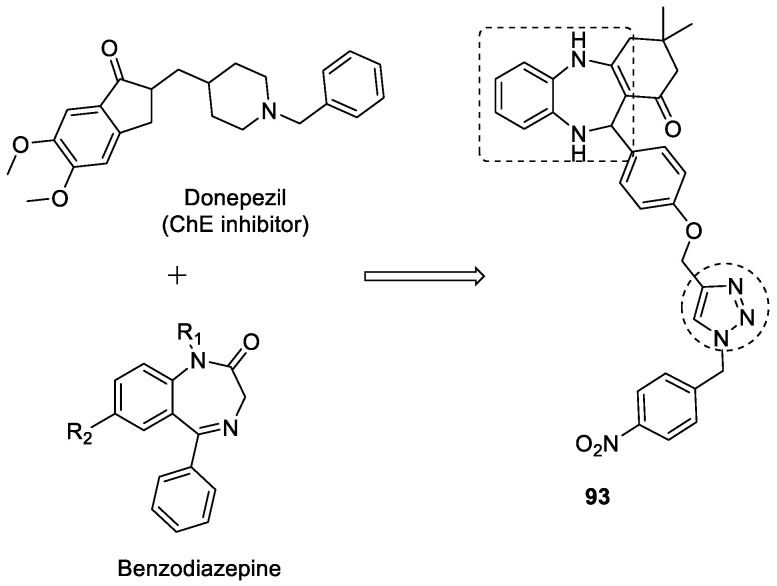
Chemical structure of benzodiazepine–1,2,3-triazole hybrid.

Benzodiazepine–1,2,3-triazole conjugate has shown more selectivity to BuChE over AChE when evaluated for ChE inhibition. The synthesized derivative 3,3-dimethyl-11-(3-((1-(4-nitrobenzyl)-1*H*-1,2,3-triazol-4-yl) methoxy) phenyl)-2,3,4,5,10,11-hexahydro-1*H*-dibenzo[*b,e*][1,4]diazepin-1-one (**93**) inhibited BuChE with an IC_50_ value of 0.2 µM (Figure 35). The **93** showed a potent inhibitory effect against Aβ self-aggregation as compared with donepezil. The BuChE inhibitory activity was supported by docking studies, as multiple interactions were observed with different active sites of the enzyme including the PAS [124].

### 3.17. Carboline–1,2,3-triazole Hybrids

**Figure 36:**
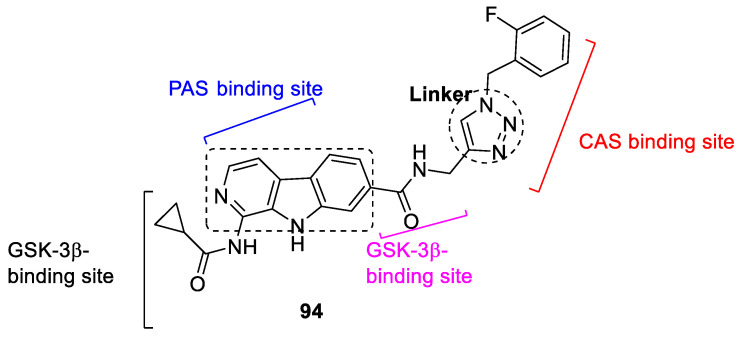
Chemical structure of carboline–1,2,3-triazole hybrid.

Novel 17-β-carboline-1,2,3-triazoles were developed as dual inhibitors of AChE glycogen synthase kinase-3β (AChE/GSK-3β) for the treatment of AD. Compound **94** showed the most potent inhibition against *ee*AChE, *h*AChE, and GSk-3β, with an IC_50_ value of 0.20 ± 0.02, 0.34 ± 0.01 and 1.14 ± 0.05 µM (Figure 36). The compound **94** showed low cytotoxicity against SH-SY5Y and HepG2 cell lines and showed diverse interaction within the binding pocket of the receptors of AChE and GSK-3β. It also inhibited the tau phosphorylation evaluated using tau (P301L) 293T cell model. The compound **94** may act as a lead for the dual inhibition of AChE and GSK-3β enzyme. [125].

### 3.18. Pyridazinone–1,2,3-triazole Hybrids

**Figure 37:**
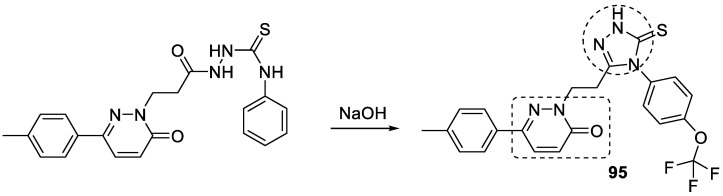
Chemical structure of pyridazinone-1,2,3-triazole hybrid.

Some novel derivatives of (*p*-tolyl)-3(2*H*)-pyridazinone with a 1,2,3-triazole moiety have been synthesized as new agents against AD. The synthesis of the 1,2,3-triazole ring takes place via the cyclization of thiosemicarbazide derivatives in the presence of NaOH. The most potent of the derivatives against AChE is a 4-trifluoromethoxy derivative **95** (Figure 37). The IC_50_ and the K*i* constant for the inhibition against AChE for compound **95** were 0.310 µM and 0.049 µM as compared with standard tacrine (IC_50_ = 0.519 and 0.226 µM (K*i*)). The molecular docking studies of the most potent compound showed a similar binding interaction as that of tacrine within the active sites [126].

### 3.19. Tryptamine–1,2,3-triazole Hybrids

**Figure 38:**
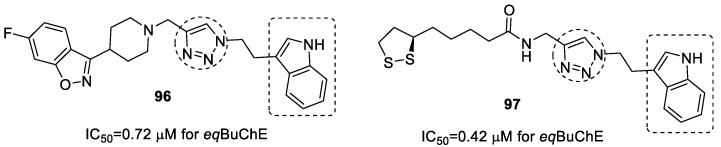
Chemical structure of tryptamine-1,2,3-triazole hybrids.

Park et al., taking a lead from a benzoisoxazoletryptamine hybrid (compound **96**), a potent BuChE inhibitor (IC_50_ = 0.72 μM) [127], designed eight new tryptamine-1,2,3-triazole hybrids. In the target compounds, which are derivatives of compound **96**, the benzoisoxazole moiety was replaced with biologically active natural compounds such as lipoic acid, polyphenolic acids (syringic acid, ferulic acid, and caffeic acid), cinnamic acid, paeonol, etc. Hybrids were prepared by reacting trytamine azide and propargylated derivatives of natural products under microwave irradiation following a click reaction. The results of in vitro anti-ChE studies showed that synthesized compounds are inactive against AChE enzyme (IC_50_ => 200 μM) but are potent and selective inhibitors of BuChE, similar to compound **96** (Figure 38). The most promising compound, **97** ((*R*)-*N*-((1-(2-(1*H*-indol-3-yl)ethyl)-1*H*-1,2,3-triazol-4-yl) methyl)-5-(1,2-dithiolan-3-yl)pentanamide), was more powerful than compound **96** (IC_50_ = 0.72 μM), and, interestingly, it was found to be a 20-fold stronger inhibitor of *eq*BuChE (IC_50_ = 0.42 μM) than galantamine (IC_50_ = 8.4 μM); however, its inhibitory activity against *h*BuChE was only 1.96 μM. Mechanistic studies showed it to be a mixed type of inhibitor that bound to PAS, CAS, anionic subsite, acyl binding pocket, and oxyanion hole on the BuChE enzyme. This compound can be used as a template to design powerful BuChE inhibitors [128].

### 3.20. Miscellaneous Hybrids of 1,2,3-triazoles

**Figure 39:**
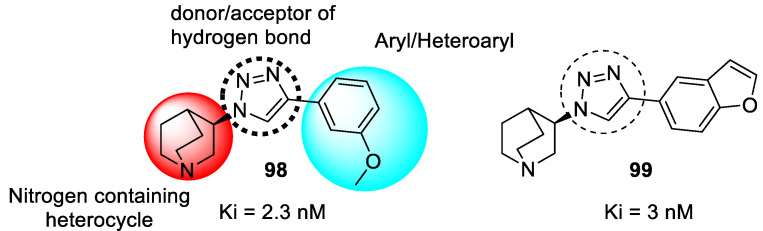
Chemical structures of quinuclidine-aryl–1,2,3-triazole hybrids.

Nicotinic agonists are of interest to treat CNS diseases, particularly schizophrenia and AD. (*R*) quinuclidine-aryl-1,2,3-triazole derivatives were synthesized and tested for in vitro α7 nicotinic ACh receptor ligands. The aryl ring includes a phenyl ring with a small methoxy; fluorine, the fluoromethyl group, or furan; thiophenes; benzofuran; and benzothiophenes heterocycles. Two compounds, **98** and **99**, showed an inhibition constant in a nanomolar range with K*i* = 2.3 and 3 nM (Figure 39). The dose range for strict selectivity toward the α4β2 nicotinic receptor was 1 µM, and these interacted with 5HT_3_ receptors with K*i* of 3 nM [129].

**Figure 40:**
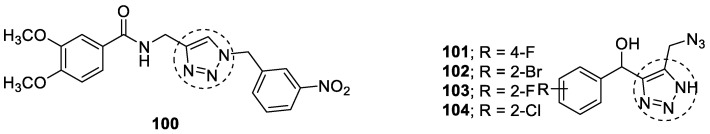
Chemical structures of miscellaneous 1,2,3-triazole hybrids.

Earlier, the promising synthesis of 1,2,3-triazole araalkylamides and their interactions within both CAS and PAS as AChE inhibitors led Petrat et al. to design and synthesize new 1,2,3-triazole araalkylamide derivatives. The synthesis takes place via a 1,3-dipolar cycloaddition reaction of propargyl amide derivatives and azide derivatives. The most potent AChE inhibitor among them was compound **100**, with an IC_50_ value of 15.01 µM (Figure 40). It was also found that compound **100** non-competitively inhibit the AChE enzyme. Aromatic residue in the structure interacted with both the CAS and PAS sites, demonstrated by the docking studies, and the 1,2,3-triazole showed van der Waals and hydrogen bond interactions with the amino acid residue in the mid-gorge region of the enzymes [130].

Anil et al. synthesized 1*H*-1,2,3-triazole using the click chemistry approach. The synthesis started with a reaction of benzaldehyde and propargyl alcohol under basic conditions to obtain 1,4-dihydroxyalkyne derivatives. The primary alcohol was converted to mesyl chloride in the presence of triethylamine and then into 1,2,3-triazole derivatives after a reaction with sodium azide. The compounds were evaluated for inhibitory activity against AChE and were found to be potent inhibitors. The IC_50_ value against AChE was in the range of 21.66–115.50 nM, and the K*i* value was in the range of 17.78 ± 3.40 to 122.57 ± 15.27 nM. Among them, compound **101**, with *para* fluoro, showed the most potent AChE inhibition, with a K*i* of 17.78 ± 3.40. The better inhibitory effect is because of the presence of halogen (Br > Cl > F) in the benzene ring. The bromine substitution showed the most pronounced effect when substituted in the order *para* > *meta* > *ortho*. The replacement of the bromine (**102**) at the second position with fluorine (**103**) and chlorine (**104**) led to a decrease in AChE inhibition by 1.60- and 1.52-fold, respectively (Figure 40) [15].

**Figure 41:**
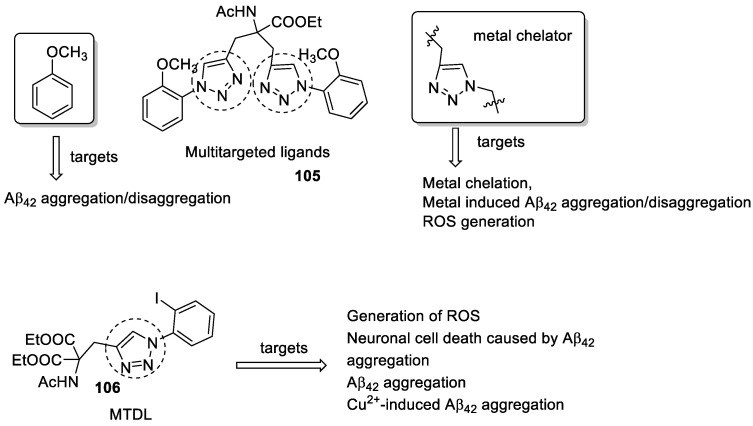
Chemical structures of miscellaneous 1,2,3-triazole hybrids.

Novel, multitargeted 1,2,3-triazole-based compounds have been synthesized against AD. The multitargets include Aβ aggregation, metal dyshomeostasis, oxidative stress, and metal-induced Aβ aggregation. Synthesized compounds with *ortho*-CF_3_ in the phenyl ring (**101**) showed the most potent inhibition against Aβ_42_ aggregation, with an IC_50_ value of 8.06 µM and 96.89% inhibition as compared with the standard curcumin, with an IC_50_ value of 6.38% and 95.14% inhibition [131]. The synthesized mono-1,2,3-triazole derivative (compound **102**) was the most potent Aβ_42_ aggregation inhibitor (IC_50_ = 4.578 µM) and showed 78.02% inhibition as compared with the standard clioquinol (CQ) (Figure 41) [132]. In addition, compounds **105** and **106** have potential metal chelating abilities, and inhibit Cu^2+^-induced Aβ_42_ aggregation. These also reduce reactive oxygen species generation (ROS) and do not display any cytotoxicity, but they were able to inhibit toxicity generated by Aβ_42_ aggregates in SH-SY5Y cells. The potent compounds **105** and **106** in molecular docking studies showed binding to the C-terminal of Aβ_42_, which plays a critical role in Aβ_42_ aggregation.

**Figure 42:**
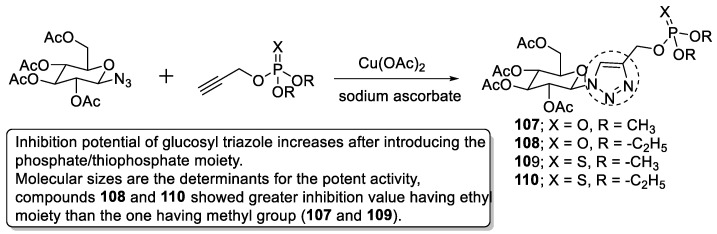
Chemical structures of miscellaneous phosphorylated/thiophosphorylated glucosyl-1,2,3-triazole hybrids.

Anjaneyulu et al. reacted propargyl alcohol with dialkoxychlorophosphate and thiophosphate derivatives to synthesize a novel series of phosphorylated/thiophosphorylated glucosyl-1,2,3-triazole derivatives. The click reaction, i.e., [2 + 3] cycloaddition via azide–alkyne, provided the final 1,2,3-triazoles derivatives (**107–110;** Figure 42). The synthesized compounds were evaluated as AChE inhibitors. Compound **106** showed the most potent AChE inhibition activity (IC_50_ = 4.56 µM) as compared with the standard paraoxon, aldicarb, carbofuran, and diisopropylfluorophosphate (DFP), with IC_50_ values of 0.86 µM, 5.32 µM, 0.516, and 5.01 µM, respectively [56].

**Figure 43:**
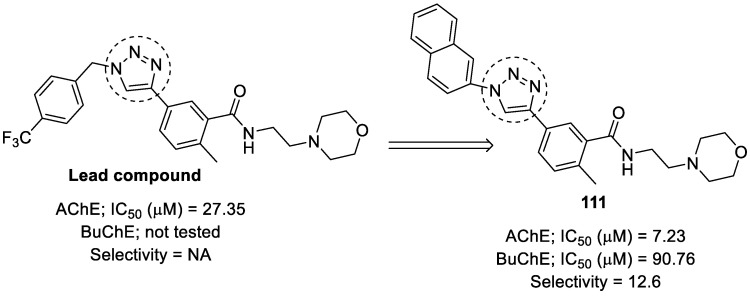
Chemical structure of a 1,2,3-triazole hybrid with AChE inhibitors and neuroprotective activity.

A new series of 1,2,3-triazole-based anti-ChE and neuroprotective compounds was designed and synthesized. As compared with the lead molecule, compound **111** showed improved AChE and BuChE inhibitory activities (IC_50_ = 7.23 and 90.76 µM, respectively) with high selectivity for AChE over BuChE (SI = 12.6). The compound showed an in vitro neuroprotective effect against Aβ_25–35_-induced neurotoxicity in SH-SY5Y cells. The hybrid molecule **111** possessed the desired physicochemical properties for CNS penetration and did not show cytotoxicity against normal cells such as human keratinocytes HaCaT and murine fibroblasts NIH-3T3. According to the preliminary results, compound **111** may be a promising dual-action treatment for AD (Figure 43) [102].

**Figure 44:**
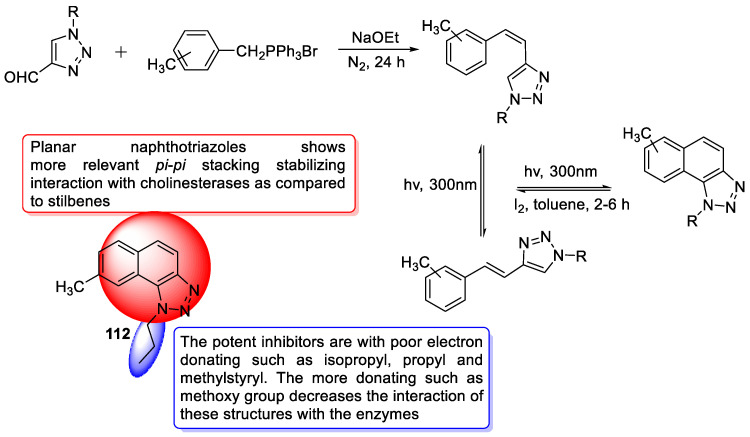
Chemical structures of miscellaneous naphtha-1,2,3-triazole hybrids.

The new substituted naphtha-1,2,3-triazoles were synthesized photochemically from new 1,2,3-triazolostilbene and evaluated for AChE and BuChE inhibition and anti-inflammatory activities. It was observed that naphtha-1,2,3-triazole photoproducts showed AChE and TNFα inhibition. Among the synthesized compounds, **112** showed the most potent *ee*AChE inhibition (IC_50_ = 671.3 µM; 78.6% inhibition) and *eq*BChE inhibition (IC_50_ = 197.8 µM; 84.3% inhibition) (Figure 44). The AChE and BuChE inhibitors also inhibited TNF-α cytokine production and demonstrated good anti-inflammatory activity [133].

**Figure 45:**
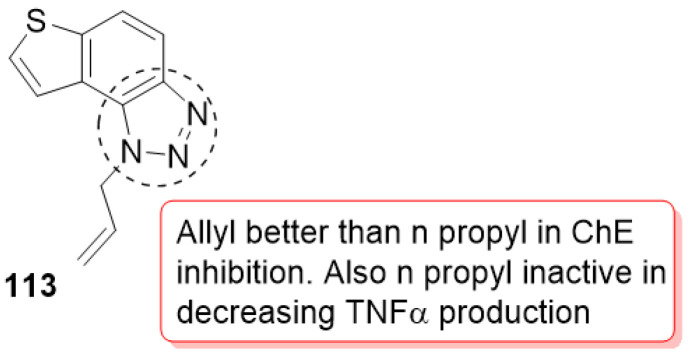
Chemical structure of a thienobenzo-1,2,3-triazole hybrid.

In an extension of the above work, Mlakic et al. synthesized thienobenzo-1,2,3-triazoles through the photochemical transformation of 1,2,3-triazolo(thieno)stilbenes and evaluated for AChE and BuChE inhibition. Among the different derivatives, allyl-thienobenzo-1,2,3-triazole (**113**) showed the most potent activity and was twice as potent as standard galantamine (IC_50_ = 7.9 µM; percentage inhibition: 90%) against *eq*BChE (IC_50_ = 3.8 µM; 80% inhibition) (Figure 45). The derivatives also showed stabilizing interactions in the active site of ChE enzymes [134].

**Figure 46:**
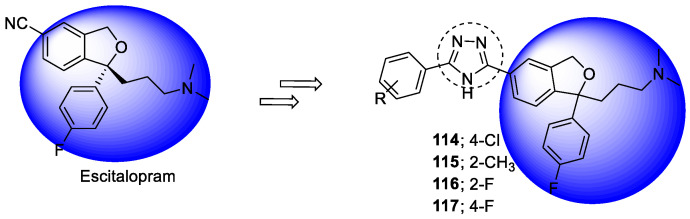
Chemical structures of miscellaneous escitalopram-1,3,4-triazole hybrids.

Escitalopram is a selective serotonin reuptake inhibitor (SSRI). A previous study reported that AD patients treated with galantamine and citalopram had improved cerebral blood flow because of the synergistic inhibition of BuChE. This led Mehr-un-Nisa et al. to synthesize novel 1,2,3-triazoles of escitalopram as ChE inhibitors. Compounds **114** (4-chlorophenyl) and **115** (2-methylphenyl) showed the highest AChE inhibition with IC_50_ values of 6.71 and 9.52 µM, whereas **116** (2-fluprophenyl) and **117** (4-fluorophenyl) showed excellent BuChE inhibition with IC_50_ values of 4.52 and 5.31 µM. It was also observed that the electron-donating group, substituted either at *ortho*, *meta*, and *para* position of the phenyl group, increased the inhibition, whereas the electron-withdrawing group decreases the inhibition. The binding energies (ΔG binds) for compounds **114** and **115** were −6.42 kcal/mol and −6.93 kcal/mol against AChE, and the binding energies of compounds **116** and **117** were −9.04 and −8.51 kcal/mol against BuChE (Figure 46) [135].

**Figure 47:**
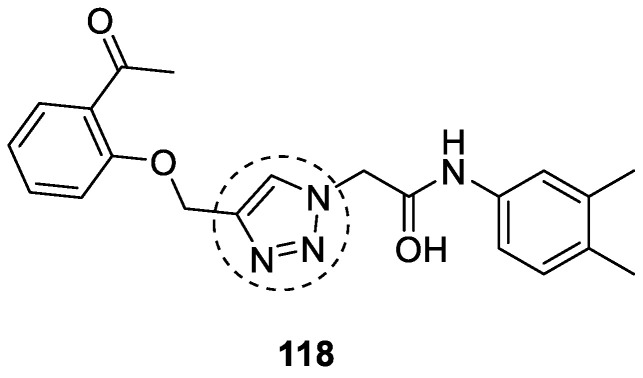
Chemical structure of a 1,2,3-triazole hybrid.

Tan and Almaz reported the synthesis and anti-ChE activity of 1,2,3-triazole derivatives derived from salicylaldehyde. The compounds were obtained in good yields following copper(I)-catalyzed azide–alkyne cycloaddition (CuAAC) reactions. The synthesized compounds showed better inhibitory activity against *ee*AChE in comparison with the *eq*BuChE enzyme. Among all the compounds, *N*-(3,4-dimethylphenyl)-2-(4-((2-formylphenoxy)methyl)-1*H*-1,2,3-triazol-1-yl)acetamide (**118**) was noted to be more powerful AChE inhibitor (IC_50_: 0.458 µM) compared with galantamine (IC_50_: 0.568 µM), but it showed weaker anti-*eq*BuChE activity (IC_50_:1.721 µM). This dual but selective AChE inhibitor (Figure 47) had a favorable pharmacokinetic profile, and in molecular docking studies, it formed binding H-bond interactions with Phe295, Tyr337, Arg296 (CAS), and Tyr124 (PAS), as well as π-π interactions (Trp86 on CAS and Tyr341 on PAS) in the active pocket of the enzyme [136].

## 4. Conclusions and Future Directions

Cholinesterase is one of the most conspicuous molecular targets for the development of pharmacotherapeutic agents to treat NDs such as AD, senile dementia, ataxia, myasthenia gravis (MG), and PD. AChE inhibitors such as donepezil, galantamine, tacrine, and rivastigmine are some of the most clinically useful anti-AD drugs. However, their usefulness is limited either due to toxicity or their inability to halt the progression of AD. Hence, medicinal chemists have used the hybridization strategy to synthesize potent dual ChE inhibitors and selective AChE or BuChE inhibitors to overcome their toxicity, improve their pharmacokinetic profiles, and develop more potent neuroprotective agents capable of acting on other molecular targets in addition to ChE inhibition. Over the past two decades, numerous hybrids of various heterocyclic moieties have been designed and synthesized as ChE inhibitors, but only one hybrid compound, ladostigil, could enter phase 3 clinical trials; however, these hybrid molecules have helped the scientific community advance the understanding of the complex pathogenesis of the disease. Furthermore, the safety, efficacy, and tolerability of ChE inhibitors entice researchers to design and develop new classes of ChE inhibitors.

One of the most widely used bioactive scaffolds is 1,2,3-triazole, which has been clubbed with one or more pharmacophoric scaffolds to develop or identify leading candidates for the treatment of NDs. The literature compiled in the current article revealed that 1,2,3-triazole has been used as a tether to connect bioactive moieties such as tacrine and coumarin, tyrosol and coumarin, coumarin and amino acids, etc. It has also been directlyconnected to the biologically active heterocyclic moieties such as quinolone, oxadiazole, indole, carbazole, pyridazinone, benzodiazepine, etc., as well as natural products including hesperetin, genipin, paeonol, tryptamine, tyrosol, etc., in hope of augmenting the anti-ChE spectrum of these scaffolds. It is interesting to note that hybrids of hesperetin, genipin tryptamine, and paeonol are selective BuChE inhibitors. Three hesperetin-1,2,3-triazole hybrids (**1, 2, 4**) and a genipin-1,2,3-triazole hybrid (**5**), 10-[4′-(7′-bromoethyl)-1*H*-1,2,3-triazole-1-yl]-1-acetoxygenipin, showed better BuChE inhibitory activity than the standard drugs donepezil and galantamine, respectively. A tryptamine–1,2,3-triazole hybrid (**97**) displayed 20-fold higher anti-BuChE activity than galantamine. However, one of the paeonol–1,2,3-triazole hybrids (**8**) was identified as the most potent and selective BuChE inhibitor (IC_50_ = 0.13 μM; SI = 769 for BuChE over AChE) among all the reported natural products and 1,2,3-triazole hybrids. It was also able to inhibit the MAO-B enzyme. Thus, it can be suggested that further structural modifications of paeonol-1,2,3-triazoles should be carried out to obtain selective BuChE inhibitors. Some of the 1,2,3-triazole hybrids, such as coumarin–1,2,3-triazole (**25, 32**), tacrine–1,2,3-triazole–coumarin trihybrids (**66**), coumarin–1,2,3-triazole (**91**), and isatin–1,2,3-triazole (**92**) hybrids, were able to inhibit AChE in a nanomolar range. Tacrine–1,2,3-triazole hybrids showed promising dual ChE inhibition, and their AChE and BuChE inhibitory activities were 3-21- and 2-140-fold more powerful than the standard drug rivastigmine (IC_50_ for AChE = 11.07 μM, and 7.72 μM against BuChE). 1*H*-1,2,3-triazole-tethered tacrine–chalcone conjugates were found to be nontoxic in comparison with tacrine. Some of the hybrid compounds displayed the desired physicochemical properties for CNS penetration and did not show cytotoxicity against normal cells. Thus, it could be concluded that 1,2,3-triazole conjugates with both synthetic and natural product scaffolds (paeonol, hesperetin, genipin) may be a viable strategy in obtaining lead compounds for the treatment of neurodegenerative diseases such as AD.

Undoubtedly, cholinergic theory is the most widely accepted hypothesis in the pathogenesis of AD, and, therefore, ChE inhibitors are considered the primary therapy for the symptomatic treatment of AD. Despite uncertainty about the duration of the benefits of clinically used ChE inhibitors, the authors believe that developing potent and selective ChE inhibitors will certainly help in delaying the progression and alleviating the symptoms associated with AD and other NDs, which may lead to better cognitive performance and quality of life for individuals. In addition, these inhibitors have been shown to exhibit disease-modifying effects. However, the paradigm in recent times is slowly shifting to developing multitarget ligands capable of acting on other targets, including ChE, to combat AD. Available clinical evidence indicates that monotherapy with ChE inhibitors is not sufficient to halt the progression or treat AD; nevertheless, small molecules developed as potent ChE inhibitors can be used in combination (as an adjunct therapy) with recently approved US FDA drugs, viz., aducanumab and leqembi™ (lecanemab-irmb), to treat AD and improve the quality of life of patients. Some of the potent compounds that are hybrids of bioactive heterocyclic moieties (isatin, indanone, tacrine, coumarin) and 1,2,3-triazole mentioned in this paper could be used as lead templates for the further optimization and development of potential ChE inhibitors as anti-AD agents.

## Figures and Tables

**Table 1:** Therapeutic actions and chemical structures of triazole-based molecules.

Pharmacological Activities	Chemical Structures
Antifungal	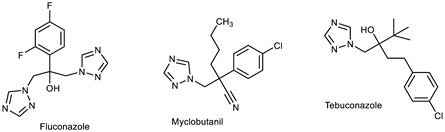
Antibiotic	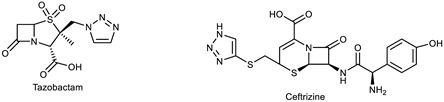
Anticancer	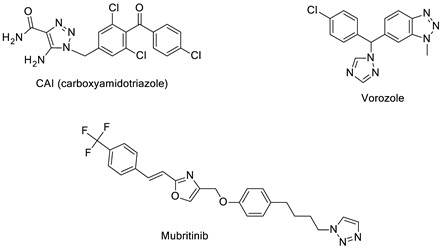
Antiviral	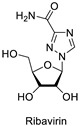
Miscellaneous	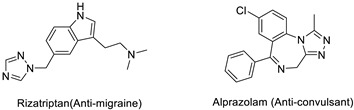

**Table 2:** Chemical structure and mechanism of the neuroprotective activities of some selected coumarin–1,2,3-triazole derivatives.

S. No	Chemical Structure	Neuroprotective Activity	Structural Features	Ref.
1	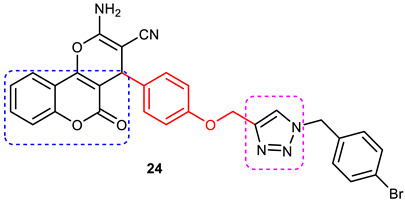	55% inhibition of AChE at 40 μg/mL.	Substitution of –Br group with a –CH_3_ or –Cl in the phenyl ring decreases activity. Introduction of –OCH_3_ group to the aryloxy moiety does not improve anti-AChE activity.	[81]
2	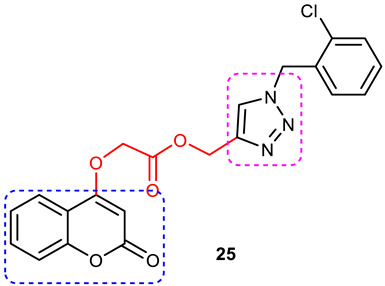	(IC_50_ = 0.18 μM-*h*AChE).	2-Cl on phenyl ring significantly improves the activity, but changing its position to *p* or replacing it with *p*-Br/*p*-NO_2_ greatly reduces anti-AChE activity (~40–250-times).	[82]
3	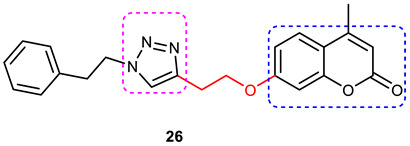	59.52% inhibition of AChE at 200 μmol/L.	The phenylethyl ring is important for exhibiting AChE inhibition	[83]
4	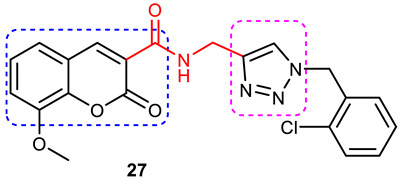	(IC_50_ = 15.42 μM)—A dual inhibitor of AChE. Protected PC12 neurons against H_2_O_2_-induced cell death.	Removal of 8-OCH_3_ group and increasing the number of chlorine atoms at 3- and 4- positions of the phenyl ring decreases AChE inhibitory activity. Unsubstituted coumarin and the phenyl ring of 1,2,3-triazole display very weak activity.	[84]
5	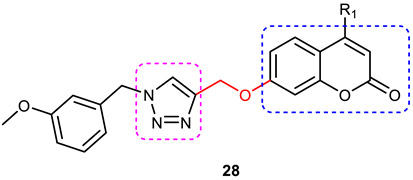	IC_50_ = 3.4 and 1.1 μM—AChE and BuChE inhibition.	2-substituted phenyl ring derivatives (Cl > F > NO_2_) are more active against AChE and BuChE than the unsubstituted derivatives.	[85]
6	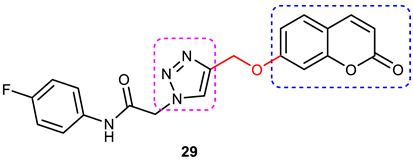	Potent AChE inhibitor (K*_i_*: 24.85 nM).	More selective toward AChE over BuChE. Acetamide group improves activity.	[86]
7	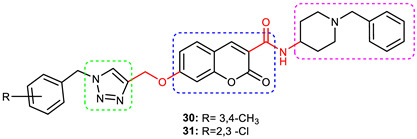	**30**: selective AChE inhibitory activity (IC_50_ = 1.80 μM). **31**: anti-BuChE activity (IC_50_ = 1.71 μM). Metal chelator.	3,4-di-methylaryl group is important for AChE inhibition. Substitution with halogen decreases AChE inhibition but enhances anti-BuChE activity.	[87]
8	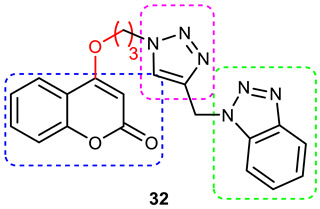	Mixed-type AChE inhibition (IC_50_ = 0.059 μΜ), copper-induced Aβ_1–42_ aggregation inhibition (34.26% at 50 μΜ).	A 3-carbon alkyl chain linker between coumarin and 1,2,3-triazole is optimal for activity. Selective AChEI, as it failed to inhibit the BuChE.	[88]
9	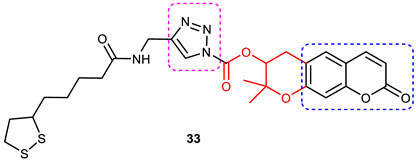	More potent BuChE inhibitor (IC_50_ = 5.89 ± 0.31 μM) than galantamine (IC_50_ = 9.4 ± 2.5 μM).	It is a decursinol hybrid which itself is not active against BuChE. Increase in activity of hybrids could be attributed to the 1,2,3-triazole–lipoic acid scaffold.	[89].
10	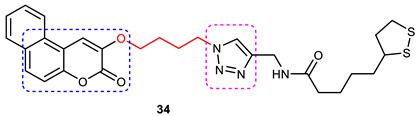	Inhibits both AChE and BuChE (IC_50_ of 7.3 and 68.6 μM).	A 4-carbon chain-linker between 1,2,3-triazole and 3-hydroxycoumarin is optimal for inhibitory activity.	[90]
11	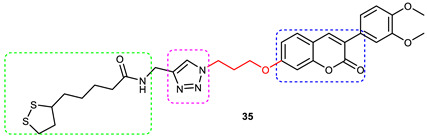	AChEI (IC_50_ = 16.4 μM)—Inhibitor of self-induced and AChE-induced Aβ_1–42_ aggregation (51.2 and 47.4%); about twofold stronger than donepezil (26 and 22.1%). Inhibits the formation of intracellular reactive oxygen species (ROS) in PC12 neuronal cells. Selective biometal chelator.	3,4-OCH_3_ phenyl at the C-3 position of a coumarin ring and linked via a three-carbon-long chain to 1,2,3-triazole is the most potent compound of the series.	[91]
12	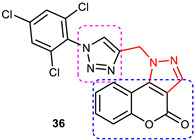	AChEI (IC_50_ = 18 μM) 5- LOX inhibitor.	The potent anti-AChE activity of this compound is attributed to the presence of three -Cl atoms on the phenyl ring.	[92]

## Data Availability

Data sharing not applicable.

## Abbreviations:

ACh	Acetylcholine
AChE	Acetylcholinesterase
*ee*AChE	eel AChE
*h*AChE	human AChE
AChEI	Acetylcholinesterase inhibitor
AD	Alzheimer’s disease
Aβ	Amyloid-β
BACE1	β-site APP cleaving enzyme-1
BBB	Blood-brain-barrier
BHT	Butylated hydroxytoluene
BuChE	Butyrylcholinesterase
*eq*BuChE	equine serum BuChE
CAS	Catalytic active (or anionic) site
ChAT	Choline acetyltransferase
ChE	Cholinesterase
CINs	Cholinergic interneurons
CuAAC	Cu(I))-catalyzed alkyne–azide 1,3-dipolar cycloaddition
DIPEA	*N*,*N*-Diisopropylethylamine or Hunig’s base
DMF	Dimethylformamide
DMSO	Dimethylsulfoxide
5HT_3_	5—hydroxy tryptamine (Serotonin)- receptor-3
LDH	Lactate dehydrogenase
LOX	Lipoxygenase
MAO	Monoamine oxidase
MG	Myasthenia gravis
MTDL	Multi-target-directed-ligand
NDs	Neurodegenerative disease
NFT	Neurofibrillary tangles
NMJ	Neuromuscular junction
NO	Nitric oxide
NT	Neurotransmitter
PAS	Peripheral anionic site
PD	Parkinson’s disease
ROS	Reactive oxygen species
SAR	Structure activity relationship
TBPB	*tert*-butyl peroxybenzoate
THF	Tetrahydrofuran
